# Racial and Ethnic Differences in Telemedicine Use

**DOI:** 10.1001/jamahealthforum.2024.0131

**Published:** 2024-03-22

**Authors:** Felippe O. Marcondes, Sharon-Lise T. Normand, Benjamin Le Cook, Haiden A. Huskamp, Jorge A. Rodriguez, Michael L. Barnett, Lori Uscher-Pines, Alisa B. Busch, Ateev Mehrotra

**Affiliations:** 1 Division of General Internal Medicine, Massachusetts General Hospital, Boston; 2 Department of Health Care Policy, Harvard Medical School, Boston, Massachusetts; 3 Department of Biostatistics, Harvard School of Public Health, Boston, Massachusetts; 4 Health Equity Research Lab, Cambridge Health Alliance, Cambridge, Massachusetts; 5 Department of Psychiatry, Harvard Medical School, Boston, Massachusetts; 6 Division of General Internal Medicine, Brigham and Women’s Hospital, Boston, Massachusetts; 7 Harvard T. H. Chan School of Public Health, Boston, Massachusetts; 8 RAND Corporation, Arlington, Virginia; 9 McLean Hospital, Belmont, Massachusetts; 10 Beth Israel Deaconess Medical Center, Boston, Massachusetts

## Abstract

**Importance:**

Individuals of racial and ethnic minority groups may be less likely to use telemedicine in part due to lack of access to technology (ie, digital divide). To date, some studies have found less telemedicine use by individuals of racial and ethnic minority groups compared with White individuals, and others have found the opposite. What explains these different findings is unclear.

**Objective:**

To quantify racial and ethnic differences in the receipt of telemedicine and total visits with and without accounting for demographic and clinical characteristics and geography.

**Design, Setting, and Participants:**

This cross-sectional study included individuals who were continuously enrolled in traditional Medicare from March 2020 to February 2022 or until death.

**Exposure:**

Race and ethnicity, which was categorized as Black non-Hispanic, Hispanic, White non-Hispanic, other (defined as American Indian/Pacific Islander, Alaska Native, and Asian), and unknown/missing.

**Main Outcomes and Measures:**

Total telemedicine visits (audio-video or audio); total visits (telemedicine or in-person) per individual during the study period. Multivariable models were used that sequentially adjusted for demographic and clinical characteristics and geographic area to examine their association with differences in telemedicine and total visit utilization by documented race and ethnicity.

**Results:**

In this national sample of 14 305 819 individuals, 7.4% reported that they were Black, 5.6% Hispanic, and 4.2% other race. In unadjusted results, compared with White individuals, Black individuals, Hispanic individuals, and individuals of other racial groups had 16.7 (95% CI, 16.1-17.3), 32.9 (95% CI, 32.3-33.6), and 20.9 (95% CI, 20.2-21.7) more telemedicine visits per 100 beneficiaries, respectively. After adjustment for clinical and demographic characteristics and geography, compared with White individuals, Black individuals, Hispanic individuals, and individuals of other racial groups had 7.9 (95% CI, −8.5 to −7.3), 13.2 (95% CI, −13.9 to −12.6), and 9.2 (95% CI, −10.0 to −8.5) fewer telemedicine visits per 100 beneficiaries, respectively. In unadjusted and fully adjusted models, and in 2019 and the second year of the COVID-19 pandemic, Black individuals, Hispanic individuals, and individuals of other racial groups continued to have fewer total visits than White individuals.

**Conclusions and Relevance:**

The results of this cross-sectional study of US Medicare enrollees suggest that although nationally, Black individuals, Hispanic individuals, and individuals of other racial groups received more telemedicine visits during the pandemic and disproportionately lived in geographic regions with higher telemedicine use, after controlling for geographic region, Black individuals, Hispanic individuals, and individuals of other racial groups received fewer telemedicine visits than White individuals.

## Key Points

**Question:**

Does telemedicine use differ by race and ethnicity?

**Finding:**

In this cross-sectional study of individuls enrolled in traditional Medicare from March 2020 to February 2022, Black and Hispanic individuals with Medicare fee-for-service insurance had higher unadjusted rates of telemedicine use compared with White individuals. After controlling for geography and demographic and clinical factors, Black and Hispanic individuals were less likely to receive telemedicine than White individuals.

**Meaning:**

The results of this study suggest that although during the COVID-19 pandemic Black and Hispanic individuals received more telemedicine visits per capita, after controlling for geographic region and other factors, Black and Hispanic individuals received less telemedicine.

## Teaser

This cross-sectional study examines racial and ethnic differences in the receipt of telemedicine and total visits with and without accounting for demographic and clinical characteristics and geography.

## Introduction

The COVID-19 pandemic was associated with a substantial increase in the use of telemedicine. With the closure of medical offices early during the pandemic, many clinicians and patients shifted to telemedicine.^1,2^ One potential advantage of this shift is that telemedicine may improve access for disadvantaged populations who face barriers to obtaining in-person care and thereby narrow long-standing disparities in the use of outpatient visits.^3^ Encouragingly, data from before the COVID-19 pandemic showed that telemedicine was preferentially used by Black and Hispanic individuals.^4^

During the pandemic, a common concern was that Black and Hispanic individuals, historically underserved racial and ethnic groups, were less likely to use telemedicine in part due to the “digital divide.” The digital divide highlights that many people lack affordable access to the necessary technology (eg, computer, smartphone, broadband internet access) and experience with the technology to have a video visit.^5^ This concern is particularly relevant to mental health treatment, given that the pandemic-associated increase in mental health symptoms and telemedicine use during the pandemic has been highest for mental health conditions.^6^

Numerous studies quantifying differences in telemedicine use during the pandemic by race and ethnicity have found conflicting findings. Many show that Black and Hispanic individuals are more likely to use telemedicine than White individuals.^5,7,8,9,10,11^ For example, a report from the US Department of Health and Human Services found that Hispanic individuals had substantially higher rates of telemedicine use than White individuals.^12^ Other studies have found the opposite, that Black and Hispanic individuals are less likely to use telemedicine than White individuals.^13,14,15,16,17^ Similarly, mixed results in telemedicine use have been found by income or other markers of socioeconomic status.^18,19^

We hypothesize that one potential explanation for these disparate findings is geography. Black and Hispanic individuals may be more likely to receive a telemedicine visit because they are more likely to live in communities with higher telemedicine use (eg, urban communities served by large health systems). However, Black and Hispanic individuals in those communities may be less likely to use telemedicine than their White neighbors. This may explain why several studies that used data from a single health system,^13,16,17^ in which patients typically come from within a limited geographic area, found lower rates of telemedicine use among Black and Hispanic individuals. The question of differences in telemedicine use by race and ethnicity and what underlies those differences is critical to guide policy interventions.

To reconcile the conflicting prior literature and address the role of geography, we used national data from the Medicare program to examine the role of clinical characteristics and geography in race and ethnicity differences in telemedicine use. In the context of this surge in telemedicine use, we also compared how the differences in overall visit counts (telemedicine plus in-person care) per person by race and ethnicity have changed from the year before the pandemic to several years into the pandemic.

## Methods

### Study Population

We used deidentified Medicare administrative claims data for a 50% random sample of all outpatient visits from March 1, 2020, to February 28, 2022. This study followed the Strengthening the Reporting of Observational Studies in Epidemiology (STROBE) reporting guideline. This study was approved by the Harvard Medical School Institutional Review Board. The first year of the pandemic covered claims from March 1, 2020, to February 28, 2021, and the second year period included claims from March 1, 2021, to February 28, 2022. We included care for all fee-for-service Medicare individuals (including dual-eligible individuals and those with a disability) who were continuously enrolled in Parts A and B during the study period or until death. Outpatient visits were based on *Current Procedural Terminology* (*CPT*) codes and place of service codes (eMethods in Supplement 1). We excluded visits in nursing homes, emergency departments, hospital inpatient facilities, or dialysis facilities because the use of telemedicine in these facility settings is often associated with a paradigm not observed in the outpatient setting (ie, clinician preference, staffing, and telemedicine technology availability).

### Measuring Visits

The primary outcome was the number of telemedicine visits per patient during the second pandemic year. We identified telemedicine visits using the relevant modifier (GT, GQ, 95) or *CPT* codes (99441-99443). The first year of the pandemic was used for collection of baseline information on covariates. The list of codes used to identify outpatient visits is provided in the eMethods in Supplement 1. As a point of comparison, we reran the analysis with the same outcomes during the first pandemic year, and the patterns were similar (eTable 1 in Supplement 1). Outlier observations could be associated with comparison between racial and ethnic groups. We addressed outliers with respect to the number of telemedicine claims by Winsorizing the number of total and telemedicine visits per patient at 52 per year. Individuals deemed outliers tended to have more comorbidities and dual-eligible status compared with nonoutliers in the analytic sample (eTable 2 in Supplement 1).

Secondary outcomes included whether an individual had any telemedicine visits (yes/no), the number of audio-only telemedicine visits (*CPT* 99441-99443), the number of telemedicine visits for mental health conditions (see the eMethods in Supplement 1 for codes that identified telemedicine mental health visits), and the number of total outpatient visits (telemedicine plus in-person) during the second pandemic year. Because of the digital divide, many have argued that audio-only visits are critical to maintaining access for disadvantaged groups.^20,21,22,23^ Several states have mandated reimbursement for audio-only visits to ensure equity.^24^ Prior work has highlighted that audio-only visits are likely undercounted in claims and electronic health records; therefore, we emphasize that the analyses focused on audio-only care should be considered exploratory.^25^

### Individual Characteristics

For each patient, we captured age (younger than 65, 65-74, 75-84, and 85 years or older), documented sex, dual Medicare and Medicaid enrollment eligibility, and zip code. Reported race and ethnicity were categorized using the Research Triangle Institute variable in Medicare’s Master Beneficiary Summary File. This variable combines self-reported data collected by the Social Security Administration and the imputation of race and ethnicity based on location and surname.^26^ Race and ethnicity in these data were categorized as American Indian/Pacific Islander, Alaska Native, Asian, non-Hispanic Black (Black), Hispanic, non-Hispanic White (White), and unknown/missing. Race and ethnicity categories of American Indian/Pacific Islander, Alaska Native, and Asian were combined into an other category due to smaller sample size and prior work highlighting that the Research Triangle Institute variable has poor sensitivity and specificity for distinguishing these racial and ethnic groups.^27^ We did not present results for the unknown/missing racial and ethnic group in the main results because it was unclear who these individuals represent. Those with unknown/missing race and ethnicity tended to be younger, male, and have fewer comorbidities compared with those with reported race and ethnicity (eTable 3 in Supplement 1).

To account for practice patterns of telemedicine use, we assigned each beneficiary to a hospital referral region (HRR) based on their zip code of residence. HRRs define tertiary health care markets based on referral patterns for major cardiovascular surgical procedures and neurosurgery.^28^ The 306 HRRs are commonly used in health services research as a better approximation for health care markets compared with states or counties.^29,30^ We obtained information on the patient’s comorbidities using the first year of the pandemic Chronic Conditions Data Warehouse Medicare Beneficiary Summary Chronic Conditions File,^31^ which includes the most common chronic conditions of Medicare patients (see eTable 4 in Supplement 1 for a complete list of comorbidities).^32^ The percentage with a high school education in the zip code was obtained from the first year of the pandemic (2020 Census).^33^

### Statistical Analyses

We summarized outcomes stratified by each of the 5 racial and ethnic groups. We also obtained the distribution by racial and ethnic groups across each HRR for each outcome. We used linear models for count outcomes and logistic models for binary outcomes to assess differences in telemedicine visits. We conducted sensitivity analyses for count outcomes using zero-inflated Poisson models because of the skew in the distribution of visit counts and because many individuals had 0 telemedicine visits. The zero-inflated Poisson models accounted for more zeros than expected and for instances in which the mean was equal to the variance. The results of the main models and sensitivity analyses were qualitatively similar (eTable 5 in Supplement 1), so we presented results from linear models.

For each outcome, the initial model included an independent variable for race and ethnicity alone (model 1). We adjusted for age, sex, and clinical indicators in a subsequent model (model 2) in concordance with the Institute of Medicine definition of disparities (ie, racial and ethnic differences in health care that are not attributed to clinical need, individual preferences, or the appropriateness of the intervention).^34^ Finally, to quantify whether the differences in visit utilization by race and ethnicity changed after accounting for where someone lived, we added a covariate for each HRR (model 3). For each outcome, we calculated the percentage rate change compared with the total population rate for each racial and ethnic group by dividing the differential number of visits (compared with White individuals) per 100 individuals by the visit rate in the total population. Recognizing that socioeconomic status may be a mediator of the association of race and ethnicity on telemedicine use, and thus should not be adjusted for, we reran the models with socioeconomic status indicators (eFigure 1 in Supplement 1) and also ran models that were restricted to dual-eligible individuals and those with a disability (eFigure 2 in Supplement 1) as sensitivity analyses.

We also conducted another analysis that restricted the cohort of Medicare individuals to those with at least 1 visit (in-person or telemedicine) during the second pandemic year. This analysis aimed to understand whether differences in telemedicine were associated with less access to any form of outpatient care. All treatment difference results from statistical analyses were accompanied with 2-sided 95% CIs and *P* values, with statistical significance defined as *P* < .05 with no adjustment for multiplicity. All statistical analyses were performed using SAS, version 9.4 (SAS Institute).

## Results

In the sample of 14 305 819 Medicare individuals, the mean (SD) age was 72.5 (11.2) years, and 7% were reported as Black, 6% as Hispanic, and 80% as White, and 4% as other race (Table 1). Also, 45.4% of individuals were male, 16.5% had Medicaid-dual enrollment, and the mean (SD) number of comorbidities was 3.2 (2.8).

**Table 1.** Population Characteristics During the Second Pandemic Period From March 2021 to February 2022^a^

Characteristic	%				
	Total (n = 14 305 819)	Black, non-Hispanic (n = 1 065 290)	Hispanic (n = 794 780)	Other, non-Hispanic (n = 603 641)^b^	White, non-Hispanic (n = 11 506 133)
Age, mean (SD), y	72.5 (11.2)	68.0 (14.0)	69.5 (13.2)	72.9 (11.3)	73.3 (10.5)
Female	54.6	55.4	53.5	57.2	55.0
Male	45.4	44.6	46.5	42.8	45.0
Medicare/Medicaid eligible	16.5	35.2	42.9	34.8	11.9
Comorbidities, mean (SD)^c^	3.2 (2.8)	3.2 (3.0)	2.9 (3.0)	3.0 (2.8)	3.2 (2.8)
Having at least a high school education (zip code level) (quintile)					
1	21.4	40.1	50.2	26.3	17.6
2	19.4	24.0	16.9	16.4	19.4
3	19.7	16.0	12.8	17.2	20.6
4	20.0	11.9	11.8	18.9	21.3
5	19.6	8.0	8.4	21.2	21.1
Census divisions					
Pacific	13.6	6.7	31.0	41.0	11.6
Mountain	7.3	2.0	10.8	7.8	7.6
West North Central	7.6	3.0	2.2	3.6	8.6
East North Central	14.5	13.2	7.0	7.7	15.4
Middle Atlantic	12.6	12.7	11.7	12.5	12.6
New England	5.6	2.4	4.1	3.3	6.1
West South Central	10.5	12.4	18.4	9.0	10.0
East South Central	6.4	10.0	1.0	1.5	6.7
South Atlantic	21.9	37.6	13.9	13.5	21.5

^a^ There were 335 975 individuals (2.3% of the total population) whose race and ethnicity category was unknown/missing (see eTable 3 in Supplement 1).

^b^ Defined as American Indian/Pacific Islander, Alaska Native, and Asian

^c^ Count of comorbidities using the Chronic Conditions Data Warehouse Medicare Beneficiary Summary Chronic Conditions File (eTable 4 in Supplement 1).

### Differences in Telemedicine Visits

**Figure 1.**
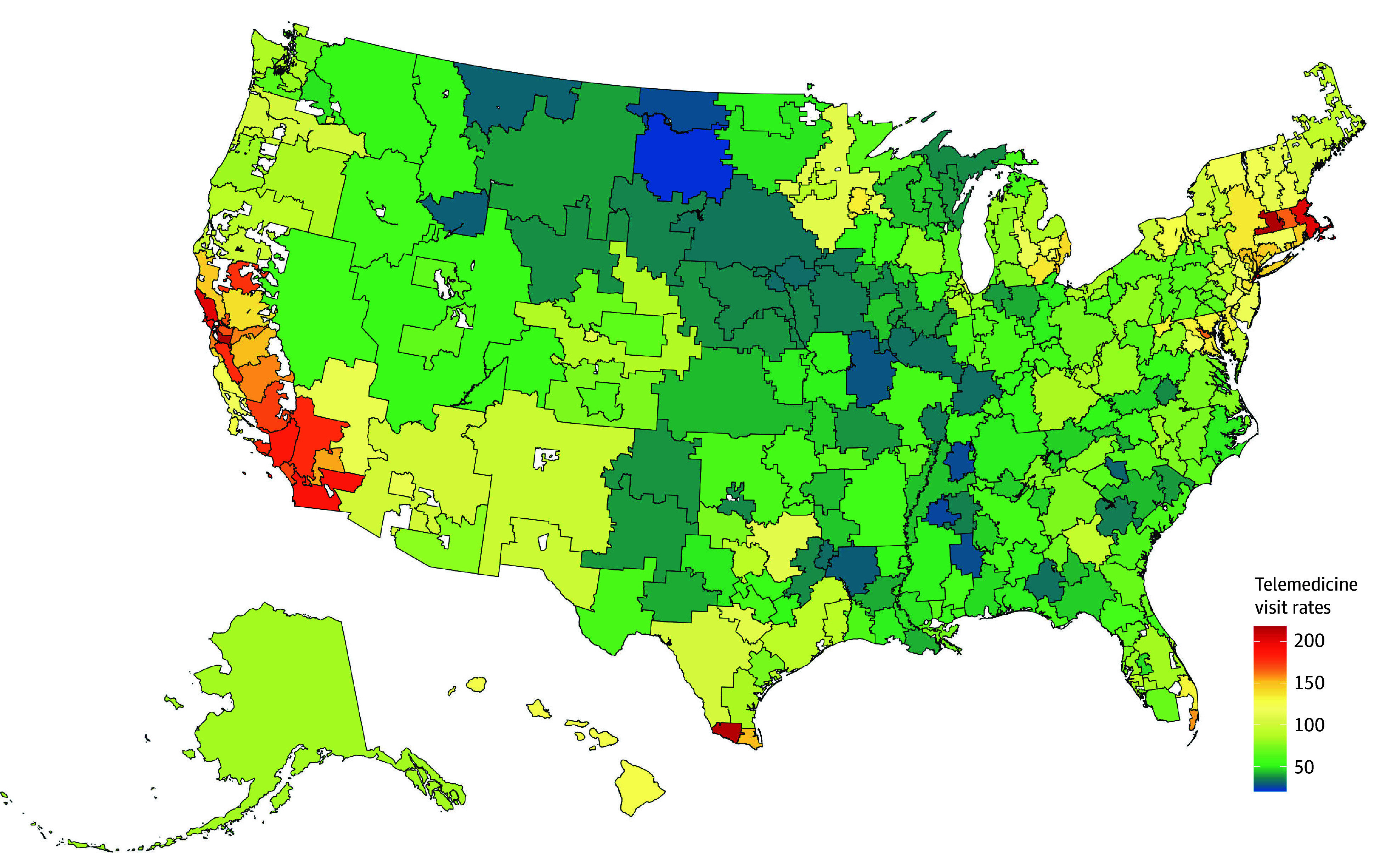
Telemedicine Visit Rates per 100 Individuals by Hospital Referral Region During the Second Pandemic Period From March 2021 to February 2022

**Figure 2.**
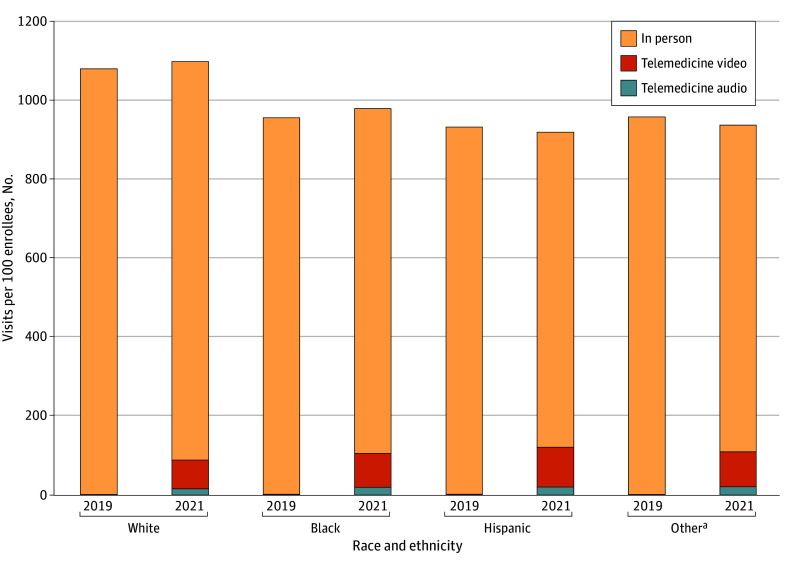
Composition of Total Outpatient Visits by Race and Ethnicity Group in 2019 and the Second Pandemic Period From March 2021 to February 2022 ^a^Data came from the full analytic sample (N = 28 724 116).

Across the population, there were 93.3 telemedicine visits per 100 individuals during the second pandemic year (March 2021-February 2022). There was significant geographic variation in telemedicine visit rates during the second year of the pandemic, with greater uptake in the Southwest and Northeast across all individuals (Figure 1; eFigure 3 in Supplement 1) and specifically for Black and Hispanic individuals (Figure 2; eFigure 4 and eTable 6 in Supplement 1) in geographic areas with higher proportions of racial and ethnic minority groups (eFigure 5 in Supplement 1).

**Figure 3.**
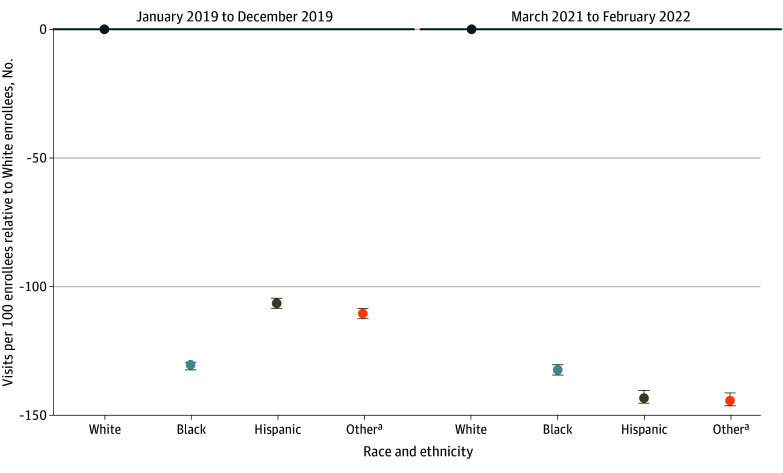
Telemedicine Visit Counts by Race and Ethnicity Group Compared With White Beneficiaries During the Second Pandemic Period From March 2021 to February 2022 ^a^Bars refer to 95% CIs.

In a model that included race and ethnicity alone (model 1; Figure 3 and Table 2), compared with White individuals, Black individuals had 16.7 (95% CI, 16.1-17.3), Hispanic individuals 32.9 (95% CI, 32.3-33.6), and individuals of other racial groups 20.9 (95% CI, 20.2-21.7) more telemedicine visits per 100 individuals. Compared with the rate of telemedicine visits in the total population, Black individuals had 17.9% (95% CI, 17.3%-18.5%), Hispanic individuals 35.3% (95% CI, 34.6%-36.0%), and individuals of other racial groups 22.4% (95% CI, 21.7%-23.3%) more telemedicine visits. After adjusting for age, sex, and clinical factors (model 2), Hispanic individuals and individuals of other racial groups continued to have more telemedicine visits compared with White individuals. The exception was that Black individuals had fewer telemedicine visits per 100 individuals (−2.4; 95% CI −3.0 to −1.8) compared with White individuals.

**Table 2.** Unadjusted and Adjusted Utilization of Telemedicine and In-Person Visits in the Second Pandemic Year From March 2021 to February 2022 as Stratified by Racial and Ethnic Group

Characteristic	Rate in total population (visits per 100 individuals)	Model	Differential visits per 100 individuals (compared with White non-Hispanic individuals)					
			Black, non-Hispanic (n = 1 065 290)	Rate change (compared with rate in total population), %	Hispanic (n = 794 780)	Rate change (compared with rate in total population), %	Other non-Hispanic (n = 603 641)^a^	Rate change (compared with rate in total population), %
Telemedicine visits^b^	93.3	Model 1^c^	16.7 (16.1 to 17.3)	17.9 (17.3 to 18.5)	32.9 (32.3 to 33.6)	35.3 (34.6 to 36.0)	20.9 (20.2 to 21.7)	22.4 (21.7 to 23.3)
		Model 2^d^	−2.4 (−3.0 to −1.8)	−2.6 (−3.2 to −1.9)	23.8 (23.2 to 24.5)	25.5 (24.9 to 26.3)	29.6 (28.8 to 30.3)	31.7 (30.9 to 32.5)
		Model 3^e^	−7.9 (−8.5 to −7.3)	−8.5 (−9.1 to −7.8)	−13.2 (−13.9 to −12.6)	−14.1 (−14.9 to −13.5	−9.2 (−10.0 to −8.5)	−9.9 (−10.7 to −9.1)
Video-only telemedicine visits^b^	77.2	Model 1	13.2 (12.6 to 13.8)	17.2 (16.3 to 17.9)	28.6 (27.9 to 29.2)	37.0 (36.1 to 37.8)	15.4 (14.7 to 16.1)	19.9 (19.0 to 20.9)
		Model 2	−3.9 (−4.5 to −3.3)	−5.1 (−5.8 to −4.3)	19.6 (18.9 to 20.2)	25.4 (24.5 to 26.2)	23.1 (22.4 to 23.9)	29.9 (29.0 to 31.0)
		Model 3	−9.8 (−10.4 to −9.3)	−12.7 (−13.5 to −12.0)	−12.9 (−13.6 to −12.6)	−16.7 (−17.6 to −16.3)	−10.8 (−11.6 to −10.1)	−14.0 (−15.0 to −13.1)
Audio-only telemedicine visits^b^	16.5	Model 1	3.5 (3.4 to 3.7)	21.2 (20.6 to 22.4)	4.3 (4.1 to 4.4)	26.1 (24.8 to 26.7)	5.4 (5.2 to 5.5)	32.7 (31.5 to 33.3)
		Model 2	1.3 (1.2 to 1.5)	7.9 (7.3 to 9.1)	4.0 (3.9 to 4.2)	24.2 (23.6 to 25.5)	6.4 (6.2 to 6.6)	38.8 (37.6 to 40.0)
		Model 3	1.6 (1.5 to 1.8)	9.7 (9.1 to 10.9)	−0.8 (−0.9 to −0.6)	−4.8 (−5.5 to −3.6)	1.3 (1.1 to 1.5)	7.9 (6.7 to 9.1)
Mental health telemedicine visits^b^	61.4	Model 1	1.0 (0.5 to 1.4)	1.6 (0.8 to 2.3)	0.8 (0.3 to 1.4)	1.3 (0.5 to 2.3)	−10.8 (−11.4 to −10.2)	−17.6 (−18.6 to −16.6)
		Model 2	−9.3 (−9.7 to −8.8)	−15.1 (−15.8 to −14.3)	−7.0 (−7.5 to −6.5)	−11.4 (−12.2 to −10.6)	−4.6 (−5.2 to −4.0)	−7.5 (−8.5 to −6.5)
		Model 3	−11.7 (−12.2 to −11.3)	−19.1 (−19.9 to −18.4)	−12.1 (−12.7 to −11.6)	−19.7 (−20.7 to −18.9)	−11.4 (−12.0 to −10.8)	−18.6 (−19.5 to −17.6)
Total visits	1073	Model 1	−119.1 (−121.2 to −117.0)	−11.1 (−11.3 to −10.9)	−174.6 (−177.0 to −172.2)	−16.3 (−16.5 to −16.0)	−156.3 (−159.1 to −153.6)	−14.6 (−14.8 to −14.3)
		Model 2	−126.2 (−128.1 to −124.3)	−11.8 (−11.9 to −11.6)	−109.7 (−111.8 to −107.5)	−10.2 (−10.4 to −10.0)	−93.8 (−96.2 to −91.4)	−8.7 (−9.0 to −8.5)
		Model 3	−165.7 (−167.6 to −163.8)	−15.4 (−15.6 to −15.3)	−186.2 (−188.4 to −184.0)	−17.4 (−17.6 to −17.1)	−163.6 (−166.1 to −161.1)	−15.2 (−15.5 to −15.0)
**In population with any visits, %**			**Odds ratio (95% CI)**					
Receipt of any telemedicine visits^f^	29.0	Model 1	1.104 (1.099 to 1.108)		1.274 (1.268 to 1.280)		1.278 (1.271 to 1.285)	
		Model 2	0.965 (0.960 to 0.969)		1.257 (1.251 to 1.264)		1.388 (1.380 to 1.396)	
		Model 3	0.916 (0.912 to 0.921)		0.856 (0.852 to 0.861)		0.931 (0.926 to 0.937)	

^a^ Defined as American Indian/Pacific Islander, Alaska Native, and Asian.

^b^ Results obtained from linear regression models.

^c^ Model 1 was defined as a model containing only the categorical variable for race and ethnicity.

^d^ Model 2 had the independent variable for racial and ethnic group and was adjusted for age, sex, and clinical factors (indicators for comorbidities).

^e^ Model 3 had the independent variable for racial and ethnic group and was adjusted for age, sex, and clinical factors (indicators for comorbidities) and included dummy variables for hospital referral region.

^f^ Results obtained from logistic regression models.

After additionally controlling for geography (model 3), compared with White individuals, all racial and ethnic minority groups had substantially fewer telemedicine visits per 100 patients (Black individuals: −7.9; 95% CI, −8.5 to −7.3; Hispanic individuals: −13.2; 95% CI, −13.9 to −12.6; individuals of other racial groups: −9.2 visits; 95% CI, −10.0 to −8.5). These differences in visits rates translated into relative differences of −8.5% (95% CI, −9.1% to −7.8%), −14.1% (95% CI, −14.9% to −13.5%), and −9.9% (95% CI, −10.7% to −9.1%) respectively for Black individuals, Hispanic individuals, and individuals of other racial groups compared with White individuals. Sensitivity analyses including dual Medicaid-Medicare eligibility status and the percentage of participants who had a high school education or more (quintiles) had similar results (eFigure 1 in Supplement 1).

We also examined whether individuals had any telemedicine visits. In unadjusted models (model 1), racial and ethnic minority groups were also more likely to have any telemedicine visits during the second pandemic year compared with White individuals (Black individuals: odds ratio [OR], 1.104; 95% CI, 1.099-1.108; Hispanic individuals: OR, 1.274; 95% CI, 1.268-1.280; individuals of other racial groups: OR, 1.278; 95% CI, 1.271-1.285). After adjusting for age, sex, and clinical factors (model 2), Black individuals were less likely to have any telemedicine visits (OR, 0.965; 95% CI, 0.960-0.969), although Hispanic and individuals of other racial groups remained more likely to have a telemedicine visit compared with White individuals. However, after fully adjusting for demographic factors, clinical factors, and geography (model 3), we found that individuals from these racial and ethnic minority groups were less likely to have any telemedicine visits (eTable 7 in Supplement 1). Sensitivity analyses of the number of telemedicine visits with zero-inflated Poisson models (eTable 5 in Supplement 1) that were restricted to individuals with at least 1 visit of any type or telemedicine visit during the second pandemic year (eTable 8 in Supplement 1) were consistent with the main analysis.

### Differences in Audio-Only Visits

Across the population there were 16.5 audio-only telemedicine visits per 100 individuals during the second pandemic year (March 2021-February 2022). In unadjusted models (model 1 in Table 2), Black individuals, Hispanic individuals, and individuals of other racial groups had more audio-only visits (3.5; 95% CI, 3.4-3.7; 4.3; 95% CI, 4.1-4.4); and 5.4 visits; 95% CI, 5.2-5.5; per 100 individuals, respectively) compared with White individuals. After adjusting for age, sex, and clinical factors (model 2), Black individuals, Hispanic individuals, and individuals of other racial groups continued having more audio-only visits compared with White individuals. In fully adjusted models controlling for demographic and clinical factors and geography (model 3), Hispanic individuals had 0.8 fewer audio-only visits (95% CI, −0.9 to −0.6) per 100 beneficiaries, while Black individuals had more audio-only visits (1.6 visits; 95% CI, 1.5-1.8) per 100 individuals compared with White individuals.

### Differences in Mental Health Visits

Across the population, there were 61.4 telemedicine visits per 100 individuals for mental health conditions. In contrast with the other outcomes, in the unadjusted models (model 1), Black individuals, Hispanic individuals, and individuals of other racial groups did not have more telemedicine visits for mental health conditions than White individuals (eTable 9 in Supplement 1). However, after adjusting for age, sex, and clinical comorbidities (model 2), Black individuals, Hispanic individuals, and individuals of other racial groups had fewer telemedicine visits for mental health conditions. In fully adjusted models (model 3), Black individuals, Hispanic individuals, and individuals of other racial groups had substantially fewer telemedicine visits for mental health conditions (−11.7; 95% CI, −12.2 to −11.3; −12.1; 95% CI, −12.7 to −11.6; −11.4; 95% CI, −12.0 to −10.8) compared with White individuals (Table 2; eTable 9 in Supplement 1). These represent relative differences of −19.1% (95% CI, −19.9% to −18.4%), −19.7% (95% CI, −20.7% to −18.9%), and −18.6% (95% CI, −19.5% to −17.6%) compared with the total population.

### Differences in Total Visits During 2019 and Second Pandemic Year

Across the total population, there were 1073 total visits per 100 individuals during the second pandemic year (March 2021-February 2022). In 2019, after adjusting for demographic and clinical characteristics and geography, the number of total visits (in-person or telemedicine) per 100 individuals were lower for Black individuals (−131.5; 95% CI, −133.0 to −130.0), Hispanic individuals (−107.2; 95% CI, −109.1 to −105.4), and individuals of other racial groups (−111.3; 95% CI, −113.4 to −109.2) compared with White individuals (eFigure 6 in Supplement 1). During the second pandemic year, these differences were similar or larger. With race and ethnicity alone in the model, the number of total visits (in-person or telemedicine) per 100 individuals were lower for Black individuals (−119.1; 95% CI, −121.2 to −117.0), Hispanic individuals (−174.6; 95% CI, −177.0 to −172.2), and individuals of other racial groups (−156.3; 95% CI, −159.1 to −153.6) compared with White individuals (eFigure 6 in Supplement 1). Similar differences were observed after adjustment for demographic characteristics and clinical factors. Black, Hispanic, and Other-race individuals continued to have fewer visits than White individuals after further adjustment for demographic and clinical factors and geography (Black individuals: −165.7; 95% CI, −167.6 to −163.8; Hispanic individuals: −186.2; 95% CI, −188.4 to −184.0; other race, −163.6; 95% CI, −166.1 to −161.1) (eFigure 6 in Supplement 1). These differences in visits rates translated into relative differences of −15.4% (95% CI, −15.6% to −15.3%), −17.4% (95% CI, −17.6% to −17.1%), and −15.2% (95% CI, −15.5% to −15.0%), respectively, for Black individuals, Hispanic individuals, and individuals of other racial groups compared with White individuals.

## Discussion

In this cross-sectional study, we found substantial differences in telemedicine use by race and ethnicity, but the direction of these differences depends on how the differences are captured. In unadjusted analyses, Black individuals, Hispanic individuals, and individuals of other racial groups had more telemedicine visits per person, and these differences largely remain even after controlling for clinical characteristics. However, after additionally controlling for geography, we found the opposite pattern, that Black individuals, Hispanic individuals, and individuals of other racial groups had fewer telemedicine visits per person. Similarly, controlling for clinical characteristics and geography, we also observed Black and Hispanic individuals had many fewer audio-only telemedicine visits and mental health telemedicine visits. To date, we did not find that there has been any narrowing of differences in overall visit counts over time.

On a national scale, our study results implied that telemedicine visit rates are higher among many individuals of racial and ethnic minority groups primarily because they are more likely to live in communities that have embraced telemedicine. This is consistent with much of the prior literature that shows an individual’s health and access to care is strongly associated to where they live.^35,36,37^

Terminology is key when discussing differences between groups of individuals. We purposefully chose to emphasize the term *differences* instead of *disparities* when comparing telemedicine use between White individuals and individuals of racial and ethnic minority groups. We acknowledge the Institute of Medicine’s definition of health care disparities as differences between racial and ethnic groups that are not attributed to clinical need, individual preferences, or appropriateness of intervention. Geography is not a component of the Institute of Medicine’s definition of health disparities, but differences due to geography could be considered to be unjust and contribute to a racial and ethnic disparity.^38^ For example, where individuals of racial and ethnic minority groups live may be associated with housing and migration patterns that reflect a history of residential segregation fueled by racism and racist public policies.^39^ However, the choice of residence may be due to personal preference and should not be treated as an unfair difference.^40^ For example, the geographic concentration of racial and ethnic groups in certain areas may represent personal preference based on work availability, climate, patterns of immigration, strong family ties to a specific location, existing ethnic enclaves, and robust social networks. As suggested in prior research,^40^ we estimated differences with and without adjustment for geography, as doing so may have implications for guiding policies that address within-region and between-region differences.^38^ In the context of this analysis, we focus on the results that were conditional on geography and believe that they highlight factors concerning differential use of telemedicine by race and ethnicity. These differences could be associated with the same factors that underlie why there is differential use of in-person visits. Unfortunately, we also did not observe any substantive change in differences in total visits per capita, a rough marker of access to care, from 2019 to the second year of the pandemic.

There has been a debate on whether greater use of audio-only visits might be associated with greater use of telemedicine observed among Black and Hispanic individuals.^17^ Recognizing substantial limitations in the capture of audio-only visits,^25^ we do not see disparate patterns for audio-only vs audio-video visits that might explain the greater use of audio-only telemedicine observed among Black individuals and individuals of other racial groups.

Telemedicine’s association with long-standing differences in utilization of care will depend on how it is implemented and to what extent an equity lens is applied to its implementation. Policies focused on racial and ethnic populations, such as generous reimbursement for telemedicine services in settings that disproportionately serve racial and ethnic minority groups, and partnerships with community organizations that host patients for their virtual appointments, investment in digital navigation programs, and creation of inclusive telemedicine platforms and workflows are some examples of interventions that could be tried and evaluated.

### Limitations

This study had limitations. First, the observational nature of the study design did not allow us to infer causation. Second, the findings described in the traditional Medicare population may not generalize to the overall population, and we do not have data on patient’s language preference, which may be an important barrier to telemedicine use. Third, as noted previously, we were limited in our ability to capture audio-only telemedicine visits. Fourth, we did not use Medicaid data, which may have provided information about use of services for those who are dually eligible for Medicare and Medicaid who were not reimbursed by Medicare. Finally, and maybe most importantly, we were limited by our data source in how we categorized individuals by race and ethnicity and were forced to aggregate many disparate groups into a single other race category.

## Conclusions

In this cross-sectional study, Black individuals, Hispanic individuals, and individuals of other racial groups with Medicare had lower outpatient telemedicine use compared with White individuals after controlling for where they live. If telemedicine is to achieve its potential to expand access to care and reduce existing disparities, federal, local, and health system policies are needed to improve digital health equity.

## Notes

Supplement 1. eMethods. Supplemental methods eTable 1. Telemedicine visits counts per 100 individuals by race and ethnicity, pandemic first year period (March 2020 to February 2021) eTable 2. Characteristics of population, pandemic second year period (March 2021 to February 2022) in analytic cohort compared to outliers Winsorized eTable 3. Cohort characteristics, pandemic second year period (March 2021 to February 2022) including individuals with Unknown/Missing race and ethnicity eTable 4. 27 Chronic Conditions Data Warehouse (CCW) Chronic Condition categories in the Medicare Beneficiary Summary File eTable 5: Telemedicine visit counts in the pandemic second year period – Sensitivity analysis with zero-inflated Poisson models eFigure 1. Telemedicine visits counts per 100 individuals by race and ethnicity group, relative to White beneficiaries, pandemic second year period - Sensitivity analysis adjusting also for measures of socioeconomic status using linear regression eFigure 2. Telemedicine visits counts per 100 individuals by race and ethnicity group, relative to White beneficiaries, pandemic second year period - Sensitivity analysis restricting population to Medicare Medicaid dual-eligible and disabled individuals eFigure 3. Composition of total outpatient visits by US census region and race and ethnicity group, 2019 and pandemic second year period (March 2021 to February 2022) eFigure 4. Telemedicine visit rates per 100 individuals by hospital referral region (HRR) by race and ethnicity, second pandemic year period (March 2021 to February 2022) eFigure 5. Proportion of individuals by hospital referral region and race and ethnicity, second pandemic year period (March 2021 to February 2022) eTable 6. Distribution of racial and ethnic groups by hospital referral region (HRR), second pandemic year period (March 2021 to February 2022) eTable 7. Odds of having a telemedicine visit (video or audio), pandemic second year period – logistic regression model results eTable 8. Telemedicine visits counts per 100 individuals by race and ethnicity, pandemic second year period – Sensitivity analysis restricting to patients with at least 1 visit in the year eTable 9. Telemedicine visits counts per 100 individuals by race and ethnicity, pandemic second year period – Sensitivity analysis restricting to mental health telemedicine visits eFigure 6. Total visit counts by race and ethnicity group, relative to White beneficiaries, 2019 and pandemic second year period (March 2021 to February 2022)

Supplement 2. Data sharing statement
