# Supplementary material for: Racial and Ethnic Differences in Telemedicine Use
Source: JAMA Health Forum. 2024 Mar 22;5(3):e240131. doi: 10.1001/jamahealthforum.2024.0131 (PMC10960201; doi:10.1001/jamahealthforum.2024.0131)
Supplement: Supplement 1. — eMethods. Supplemental methods eTable 1. Telemedicine visits counts per 100 individuals by race and ethnicity, pandemic first year period (March 2020 to February 2021) eTable 2. Characteristics of population, pandemic second year period (March 2021 to February 2022) in analytic cohort compared to outliers Winsorized eTable 3. Cohort characteristics, pandemic second year period (March 2021 to February 2022) including individuals with Unknown/Missing race and ethnicity eTable 4. 27 Chronic Conditions Data Warehouse (CCW) Chronic Condition categories in the Medicare Beneficiary Summary File eTable 5: Telemedicine visit counts in the pandemic second year period – Sensitivity analysis with zero-inflated Poisson models eFigure 1. Telemedicine visits counts per 100 individuals by race and ethnicity group, relative to White beneficiaries, pandemic second year period - Sensitivity analysis adjusting also for measures of socioeconomic status using linear regression eFigure 2. Telemedicine visits counts per 100 individuals by race and ethnicity group, relative to White beneficiaries, pandemic second year period - Sensitivity analysis restricting population to Medicare Medicaid dual-eligible and disabled individuals eFigure 3. Composition of total outpatient visits by US census region and race and ethnicity group, 2019 and pandemic second year period (March 2021 to February 2022) eFigure 4. Telemedicine visit rates per 100 individuals by hospital referral region (HRR) by race and ethnicity, second pandemic year period (March 2021 to February 2022) eFigure 5. Proportion of individuals by hospital referral region and race and ethnicity, second pandemic year period (March 2021 to February 2022) eTable 6. Distribution of racial and ethnic groups by hospital referral region (HRR), second pandemic year period (March 2021 to February 2022) eTable 7. Odds of having a telemedicine visit (video or audio), pandemic second year period – logistic regression model results eTable 8. Telemedic [file jamahealthforum-e240131-s001.pdf]

Supplemental Online Content

Marcondes FO, Normand S-L T, Le Cook B, et al. Racial and ethnic differences in telemedicine use. *JAMA Health Forum*. 2024;5(3):e240131. doi:10.1001/jamahealthforum.2024.0131

eMethods. Supplemental methods

eTable 1. Telemedicine visits counts per 100 individuals by race and ethnicity, pandemic first year period (March 2020 to February 2021)

eTable 2. Characteristics of population, pandemic second year period (March 2021 to February 2022) in analytic cohort compared to outliers Winsorized

eTable 3. Cohort characteristics, pandemic second year period (March 2021 to February 2022) including individuals with Unknown/Missing race and ethnicity

eTable 4. 27 Chronic Conditions Data Warehouse (CCW) Chronic Condition categories in the Medicare Beneficiary Summary File

eTable 5: Telemedicine visit counts in the pandemic second year period – Sensitivity analysis with zero-inflated Poisson models

eFigure 1. Telemedicine visits counts per 100 individuals by race and ethnicity group, relative to White beneficiaries, pandemic second year period - Sensitivity analysis adjusting also for measures of socioeconomic status using linear regression

eFigure 2. Telemedicine visits counts per 100 individuals by race and ethnicity group, relative to White beneficiaries, pandemic second year period - Sensitivity analysis restricting population to Medicare Medicaid dual-eligible and disabled individuals

eFigure 3. Composition of total outpatient visits by US census region and race and ethnicity group, 2019 and pandemic second year period (March 2021 to February 2022)

eFigure 4. Telemedicine visit rates per 100 individuals by hospital referral region (HRR) by race and ethnicity, second pandemic year period (March 2021 to February 2022)

eFigure 5. Proportion of individuals by hospital referral region and race and ethnicity, second pandemic year period (March 2021 to February 2022)

eTable 6. Distribution of racial and ethnic groups by hospital referral region (HRR), second pandemic year period (March 2021 to February 2022)

eTable 7. Odds of having a telemedicine visit (video or audio), pandemic second year period – logistic regression model results

eTable 8. Telemedicine visits counts per 100 individuals by race and ethnicity, pandemic second year period – Sensitivity analysis restricting to patients with at least 1 visit in the year

eTable 9. Telemedicine visits counts per 100 individuals by race and ethnicity, pandemic second year period – Sensitivity analysis restricting to mental health telemedicine visits

eFigure 6. Total visit counts by race and ethnicity group, relative to White beneficiaries, 2019 and pandemic second year period (March 2021 to February 2022)

© 2024 Marcondes FO et al. *JAMA Health Forum.*

This supplemental material has been provided by the authors to give readers additional information about their work.

© 2024 Marcondes FO et al. *JAMA Health Forum.*

eMethods: Supplemental Methods

Classification of Outpatient Visits

We defined outpatient visits using Medicare's expanded list of outpatient Common Procedural Terminology (CPT) codes. We excluded all CPT codes that were specific for clinical settings outside of clinician offices (e.g., emergency departments, hospital inpatient, nursing home or dialysis facility codes). Below is the list of CPT codes specific to outpatient care that were included in our analysis.

CPT	Short Description	CPT	Short Description
77427	Radiation tx management x5	97155	Adapt behavior tx phys/qhp
90785	Psytx complex interactive	97156	Fam adapt bhv tx gdn phy/qhp
90791	Psych diagnostic evaluation	97157	Mult fam adapt bhv tx gdn
90792	Psych diag eval w/med srvcs	97158	Grp adapt bhv tx by phy/qhp
90832	Psytx w pt 30 minutes	97161	Pt eval low complex 20 min
90833	Psytx w pt w e/m 30 min	97162	Pt eval mod complex 30 min
90834	Psytx w pt 45 minutes	97163	Pt eval high complex 45 min
90836	Psytx w pt w e/m 45 min	97164	Pt re-eval est plan care
90837	Psytx w pt 60 minutes	97165	Ot eval low complex 30 min
90838	Psytx w pt w e/m 60 min	97166	Ot eval mod complex 45 min
90839	Psytx crisis initial 60 min	97167	Ot eval high complex 60 min
90840	Psytx crisis ea addl 30 min	97168	Ot re-eval est plan care
90845	Psychoanalysis	97530	Therapeutic activities
90846	Family psytx w/o pt 50 min	97535	Self-care mngment training
90847	Family psytx w/pt 50 min	97542	Wheelchair mngment training
90853	Group psychotherapy	97750	Physical performance test
90875	Psychophysiological therapy	97755	Assistive technology assess
90951	ESRD serv 4 visits p mo <2yr	97760	Orthotic mgmt&traing 1st enc
90952	ESRD serv 2-3 vsts p mo <2yr	97761	Prosthetic traing 1st enc
90953	ESRD serv 1 visit p mo <2yrs	97802	Medical nutrition indiv in
90954	ESRD serv 4 vsts p mo 2-11	97803	Med nutrition indiv subseq
90955	ESRD srv 2-3 vsts p mo 2-11	97804	Medical nutrition group
90956	ESRD srv 1 visit p mo 2-11	99201	Office/outpatient visit new
90957	ESRD srv 4 vsts p mo 12-19	99202	Office/outpatient visit new
90958	ESRD srv 2-3 vsts p mo 12-19	99203	Office/outpatient visit new
90959	ESRD serv 1 vst p mo 12-19	99204	Office/outpatient visit new
90960	ESRD srv 4 visits p mo 20+	99205	Office/outpatient visit new
90961	ESRD srv 2-3 vsts p mo 20+	99211	Office/outpatient visit est
90962	ESRD serv 1 visit p mo 20+	99212	Office/outpatient visit est
90963	ESRD home pt serv p mo <2yrs	99213	Office/outpatient visit est
90964	ESRD home pt serv p mo 2-11	99214	Office/outpatient visit est
90965	ESRD home pt serv p mo 12-19	99215	Office/outpatient visit est
90966	ESRD home pt serv p mo 20+	99354	Prolong e&m/psyctx serv o/p
90967	ESRD svc pr day pt <2	99355	Prolong e&m/psyctx serv o/p
90968	ESRD svc pr day pt 2-11	99406	Behav chng smoking 3-10 min
90969	ESRD svc pr day pt 12-19	99407	Behav chng smoking > 10 min
90970	ESRD svc pr day pt 20+	99441	Phone e/m phys/qhp 5-10 min
92002	Eye exam new patient	99442	Phone e/m phys/qhp 11-20 min
92004	Eye exam new patient	99443	Phone e/m phys/qhp 21-30 min
92012	Eye exam establish patient	99473	Self-meas bp pt educaj/train
92014	Eye exam&tx estab pt 1/>vst	99483	Assmt & care pln pt cog imp
92507	Speech/hearing therapy	99495	Trans care mgmt 14 day disch
92508	Speech/hearing therapy	99496	Trans care mgmt 7 day disch
92521	Evaluation of speech fluency	99497	Advncd care plan 30 min
92522	Evaluate speech production	99498	Advncd care plan addl 30 min
92523	Speech sound lang comprehen	0373T	Adapt bhv tx ea 15 min
92524	Behavral qualit analys voice	S9152	Speech therapy, re-eval
92601	Cochlear implt f/up exam <7	0362T	Bhv id suprt assmt ea 15 min
92602	Reprogram cochlear implt <7	G0108	Diab manage trn per indiv

© 2024 Marcondes FO et al. *JAMA Health Forum.*

92603	Cochlear implt f/up exam 7/>
92604	Reprogram cochlear implt 7/>
94005	Home vent mgmt supervision
94664	Evaluate pt use of inhaler
96110	Developmental screen w/score
96112	Devel tst phys/qhp 1st hr
96113	Devel tst phys/qhp ea addl
96116	Nubhvl xm phys/qhp 1st hr
96121	Nubhvl xm phy/qhp ea addl hr
96127	Brief emotional/behav assmt
96130	Psycl tst eval phys/qhp 1st
96131	Psycl tst eval phys/qhp ea
96132	Nrpsyc tst eval phys/qhp 1st
96133	Nrpsyc tst eval phys/qhp ea
96136	Psycl/nrpsyc tst phy/qhp 1st
96137	Psycl/nrpsyc tst phy/qhp ea
96138	Psycl/nrpsyc tech 1st
96139	Psycl/nrpsyc tst tech ea
96156	Hlth bhv assmt/reassessment
96158	Hlth bhv ivntj indiv 1st 30
96159	Hlth bhv ivntj indiv ea addl
96160	Pt-focused hlth risk assmt
96161	Caregiver health risk assmt
96164	Hlth bhv ivntj grp 1st 30
96165	Hlth bhv ivntj grp ea addl
96167	Hlth bhv ivntj fam 1st 30
96168	Hlth bhv ivntj fam ea addl
96170	Hlth bhv ivntj fam wo pt 1st
96171	Hlth bhv ivntj fam w/o pt ea
97110	Therapeutic exercises
97112	Neuromuscular reeducation
G0109	Diab manage trn ind/group
G0270	Mnt subs tx for change dx
G0296	Visit to determ ldct elig
G0396	Alcohol/subs interv 15-30mn
G0397	Alcohol/subs interv >30 min
G0410	Grp psych partial hosp 45-50
G0420	Ed svc ckd ind per session
G0421	Ed svc ckd grp per session
G0436	Tobacco-use counsel 3-10 min
G0437	Tobacco-use counsel>10min
G0438	Ppps, initial visit
G0439	Ppps, subseq visit
G0442	Annual alcohol screen 15 min
G0443	Brief alcohol misuse counsel
G0444	Depression screen annual
G0445	High inten beh couns std 30m
G0446	Intens behave ther cardio dx
G0447	Behavior counsel obesity 15m
G0506	Comp asses care plan ccm svc
G0513	Prolong prev svcs, first 30m
G0514	Prolong prev svcs, addl 30m
G2086	Off base opioid tx 70min
G2087	Off base opioid tx, 60 m
G2088	Off base opioid tx, add30
97116	Gait training therapy
97150	Group therapeutic procedures
97151	Bhv id assmt by phys/qhp
97152	Bhv id suprt assmt by 1 tech
97153	Adaptive behavior tx by tech
97154	Grp adapt bhv tx by tech

Identification of outpatient visits for mental health

Outpatient mental health visits were defined as:

Any of the Current Procedural Terminology (CPT) codes for ambulatory/outpatient visits as noted above

And

A primary diagnosis code for a mental illness for the visit as ICD-10: F20-F69, F80-F89 (developmental disorders including autism), F90 –F98 (childhood onset disorders), F99 (unspecified mental disorder)

© 2024 Marcondes FO et al. *JAMA Health Forum.*

eTable 1. Telemedicine visits counts per 100 individuals by race and ethnicity, pandemic first year period (March 2020 to February 2021)

	Telemedicine (audio-video) visits (%)		
	Estimate (95% CI)		
**Race/ethnicity**			
White	Ref	Ref	Ref
Black	11.0 (10.4, 11.7)	-8.9 (-9.5, -8.3)	-15.3 (-15.9, -14.7)
Hispanic	33.0 (32.2, 33.7)	25.9 (25.2, 26.6)	-14.2 (-15.0, -13.5)
Other	11.6 (10.8, 12.4)	25.7 (24.9, 26.5)	-13.5 (-14.3, -12.6)
**Regression model**	Model 1<br><br>No other variables	Model 2<br><br>Age, Documented sex, Clinical indicators	Model 3<br><br>Adding indicator variables for HRR[a]

a Abbrev: HRR - Hospital Referral Region

© 2024 Marcondes FO et al. *JAMA Health Forum.*

eTable 2. Characteristics of population, pandemic second year period (March 2021 to February 2022) in analytic cohort compared to outliers Winsorized

Cohort	Main analytic cohort (n=14,305,819)	Outliers[a] (n=4,198,310)
Age (mean) (SD)	72.5 (11.2)	71.7 (12.1)
Male	45.4	40.8
Medicare/Medicaid eligible	16.5	22.5
Comorbidities[b] (mean) (SD)	3.2 (2.8)	4.1 (2.9)
Percent having at least high school education (ZIP code level) (quintile)		
1	21.4	21.2
2	19.4	18.0
3	19.7	18.8
4	20.0	20.3
5	19.6	21.7
Census divisions		
Pacific	13.6	18.4
Mountain	7.3	6.8
West North Central	7.6	4.8
East North Central	14.5	12.5
Middle Atlantic	12.6	14.4
New England	5.6	7.5
West South Central	10.5	9.9
East South Central	6.4	4.6
South Atlantic	21.9	21.1

a Outliers were defined as having either total visits or telemedicine visits greater than 52 in 2021. We addressed outliers in telemedicine claims by Winsorizing (capping) the number of visits at 52 per year.b Count of comorbidities using the Chronic Conditions Data Warehouse Medicare Beneficiary Summary Chronic Conditions File. Full list provided in Supplement.

© 2024 Marcondes FO et al. *JAMA Health Forum.*

eTable 3. Cohort characteristics, pandemic second year period (March 2021 to February 2022) including individuals with Unknown/Missing race and ethnicity

**Cohort**	Overall N=14,305,819 %	White, non-Hispanic N=11,506,133 %	Black, non-Hispanic N=1,065,290 %	Hispanic N=794,780 %	Other non-Hispanic N=603,641 %	Unknown and Missing N=335,975 %
Age (mean) (SD)	72.5 (11.2)	73.3 (10.5)	68.0 (14.0)	69.5 (13.2)	72.9 (11.3)	67.4 (12.6)
Male	45.4	45.0	44.6	46.5	42.8	62.6
Medicare/Medicaid eligible	16.5	11.9	35.2	42.9	34.8	16.5
Comorbidities (mean) (SD)	3.2 (2.8)	3.2 (2.8)	3.2 (3.0)	2.9 (3.0)	3.0 (2.8)	2.4 (2.4)
Percent having at least high school education (ZIP code level) (quintile)						
1	21.4	17.6	40.1	50.2	26.3	13.6
2	19.4	19.4	24.0	16.9	16.4	16.0
3	19.7	20.6	16.0	12.8	17.2	19.3
4	20.0	21.3	11.9	11.8	18.9	22.4
5	19.6	21.1	8.0	8.4	21.2	28.7

© 2024 Marcondes FO et al. *JAMA Health Forum.*

eTable 4. 27 Chronic Conditions Data Warehouse (CCW) Chronic Condition categories in the Medicare Beneficiary Summary File

Acquired Hypothyroidism
Acute Myocardial Infarction
Alzheimer's Disease
Alzheimer's Disease and Related Disorders or Senile Dementia
Anemia
Asthma
Atrial Fibrillation
Benign Prostatic Hyperplasia
Cancer, Breast
Cancer, Colorectal
Cancer, Endometrial
Cancer, Lung
Cancer, Prostate
Cataract
Chronic Kidney Disease
Chronic Obstructive Pulmonary Disease and Bronchiectasis
Depression
Diabetes
Glaucoma
Heart Failure
Hip/Pelvic Fracture
Hyperlipidemia
Hypertension
Ischemic Heart Disease
Osteoporosis
Rheumatoid Arthritis/Osteoarthritis
Stroke/Transient Ischemic Attack

© 2024 Marcondes FO et al. *JAMA Health Forum.*

eTable 5. Telemedicine visit counts in the pandemic second year period – Sensitivity analysis with zero-inflated Poisson models

	Telemedicine (audio-video) visits (%)					
	Estimate (95% CI)					
Race/ethnicity	Poisson Count Model[a]	Excess Zero Model[b]	Poisson Count Model	Excess Zero Model	Poisson Count Model	Excess Zero Model
White	Ref	Ref	Ref	Ref	Ref	Ref
Black	1.126 (1.123, 1.128)	1.080 (1.075, 1.084)	1.022 (1.020, 1.024)	0.954 (0.949, 0.958)	0.983 (0.981, 0.986)	0.914 (0.909, 0.918)
Hispanic	1.184 (1.181, 1.186)	1.239 (1.232, 1.245)	1.098 (1.096, 1.101)	1.228 (1.222, 1.235)	0.932 (0.930, 0.935)	0.871 (0.866, 0.876)
Other	1.050 (1.047, 1.053)	1.271 (1.264, 1.278)	1.161 (1.158, 1.165)	1.335 (1.326, 1.343)	0.989 (0.986, 0.992)	0.928 (0.922, 0.934)
Regression model	Model 1<br><br>No other variables		Model 2<br><br>Age, Documented sex, Clinical indicators		Model 3<br><br>Adding indicator variables for HRR[c]	

a Poisson Count Model columns represent incidence rate ratios

b Excess Zero Model columns represent odds ratios

c Abbrev: HRR - Hospital Referral Region

© 2024 Marcondes FO et al. *JAMA Health Forum.*

eFigure 1. Telemedicine visits counts per 100 individuals by race and ethnicity group, relative to White beneficiaries, pandemic second year period - Sensitivity analysis adjusting also for measures of socioeconomic status using linear regression

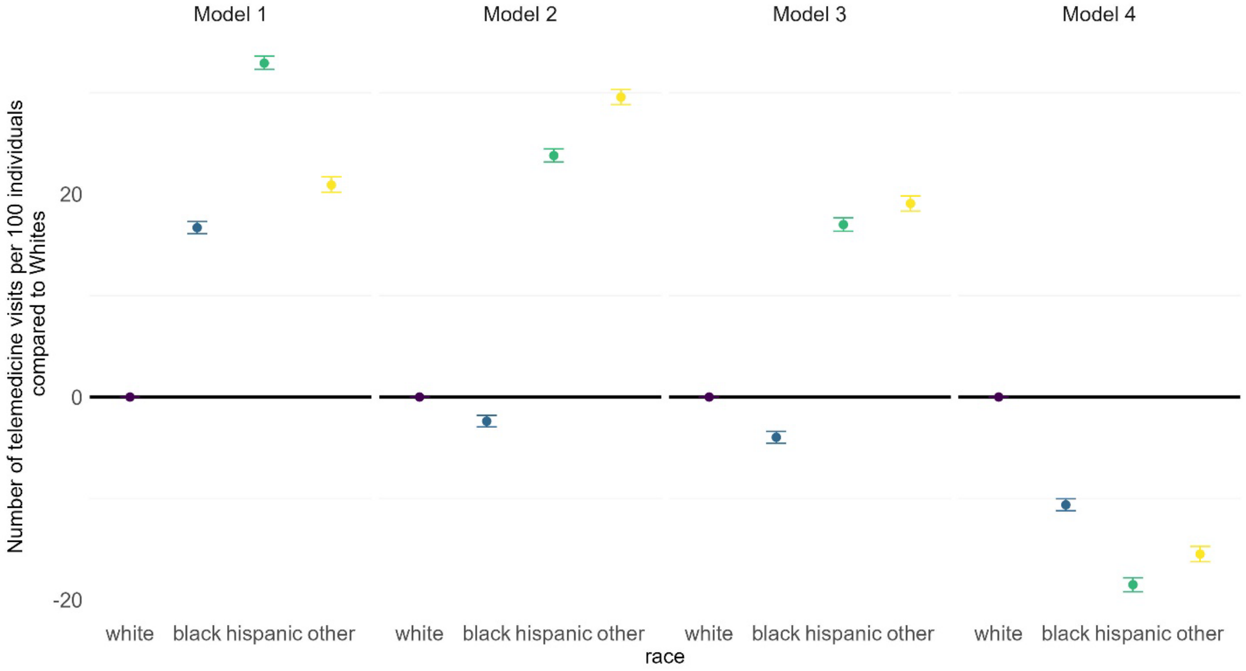

Regression model	Model 1	Model 2	Model 3	Model 4
	No other variables	Age, documented sex, clinical indicators	Adding Medicaid dual status, ZIP code-level % having high school education (quintile)	Adding indicator variables for HRR

© 2024 Marcondes FO et al. *JAMA Health Forum.*

eFigure 2. Telemedicine visits counts per 100 individuals by race and ethnicity group, relative to White beneficiaries, pandemic second year period - Sensitivity analysis restricting population to Medicare Medicaid dual-eligible and disabled individuals

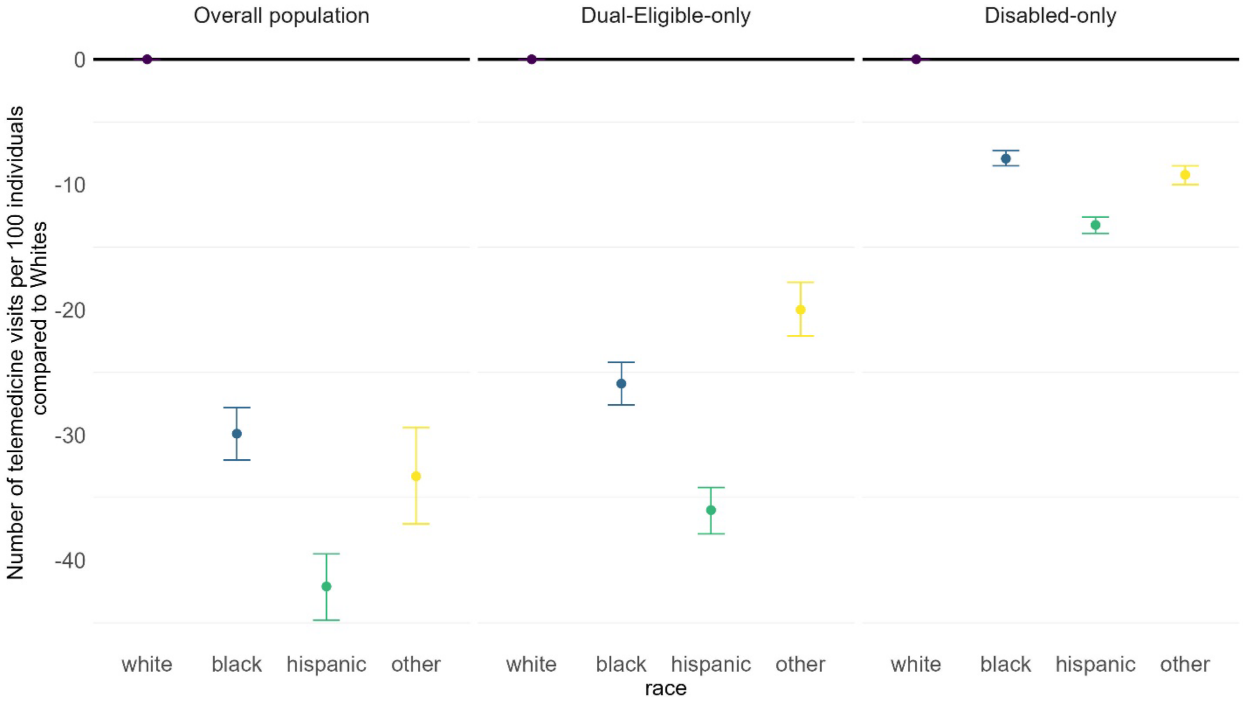

Regression model	Model 3<br><br>Age, documented sex, clinical indicators, indicator variables for HRR	Model 3<br><br>Age, documented sex, clinical indicators, indicator variables for HRR	Model 3<br><br>Age, documented sex, clinical indicators, indicator variables for HRR

© 2024 Marcondes FO et al. *JAMA Health Forum.*

eFigure 3. Outpatient visits by race and ethnicity, 2019 to second pandemic year period (March 2021 to February 2022)[a,b]

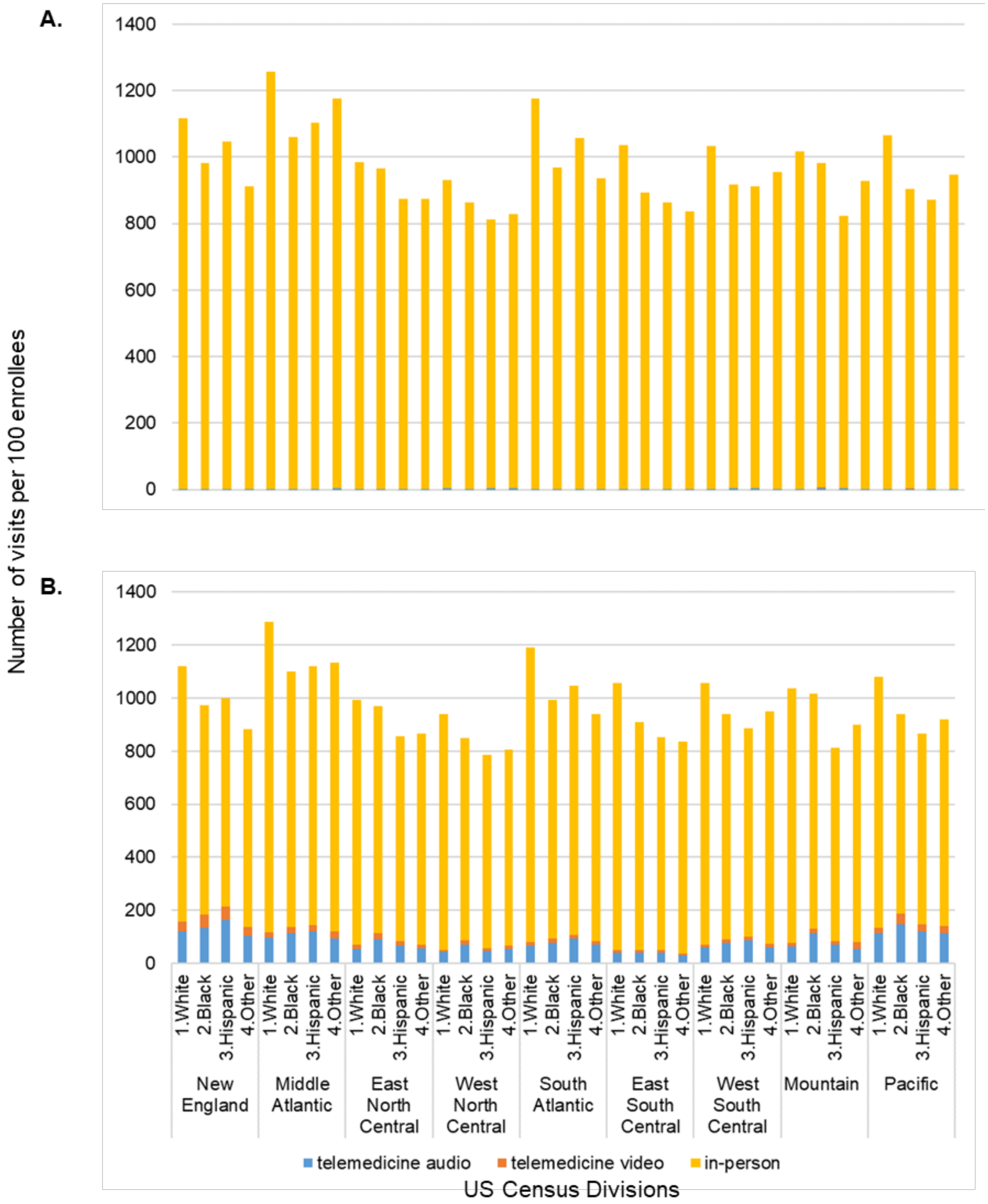

a Panel A denotes calendar year 2019 and Panel B denotes second pandemic year period (covered claims from March 1, 2021, to February 28, 2022).

b Note: Data for figure came from 100% of the analytic sample (N = 28,724,116).

© 2024 Marcondes FO et al. *JAMA Health Forum.*

eFigure 4. Telemedicine visit rates per 100 individuals by hospital referral region by race and ethnicity, second pandemic year period (March 2021 to February 2022)

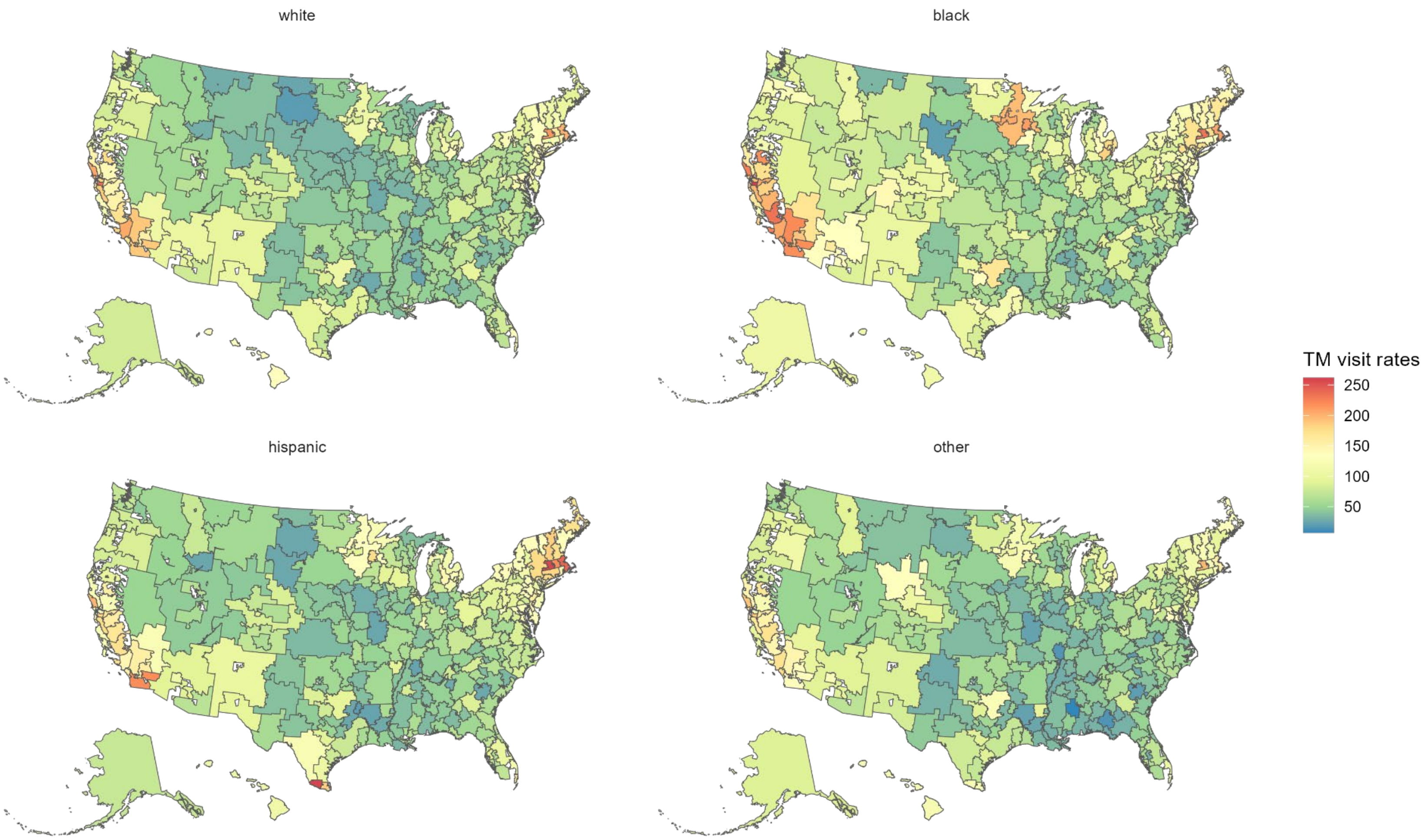

© 2024 Marcondes FO et al. *JAMA Health Forum.*

eFigure 5. Proportion of individuals by hospital referral region and race and ethnicity, second pandemic year period (March 2021 to February 2022)

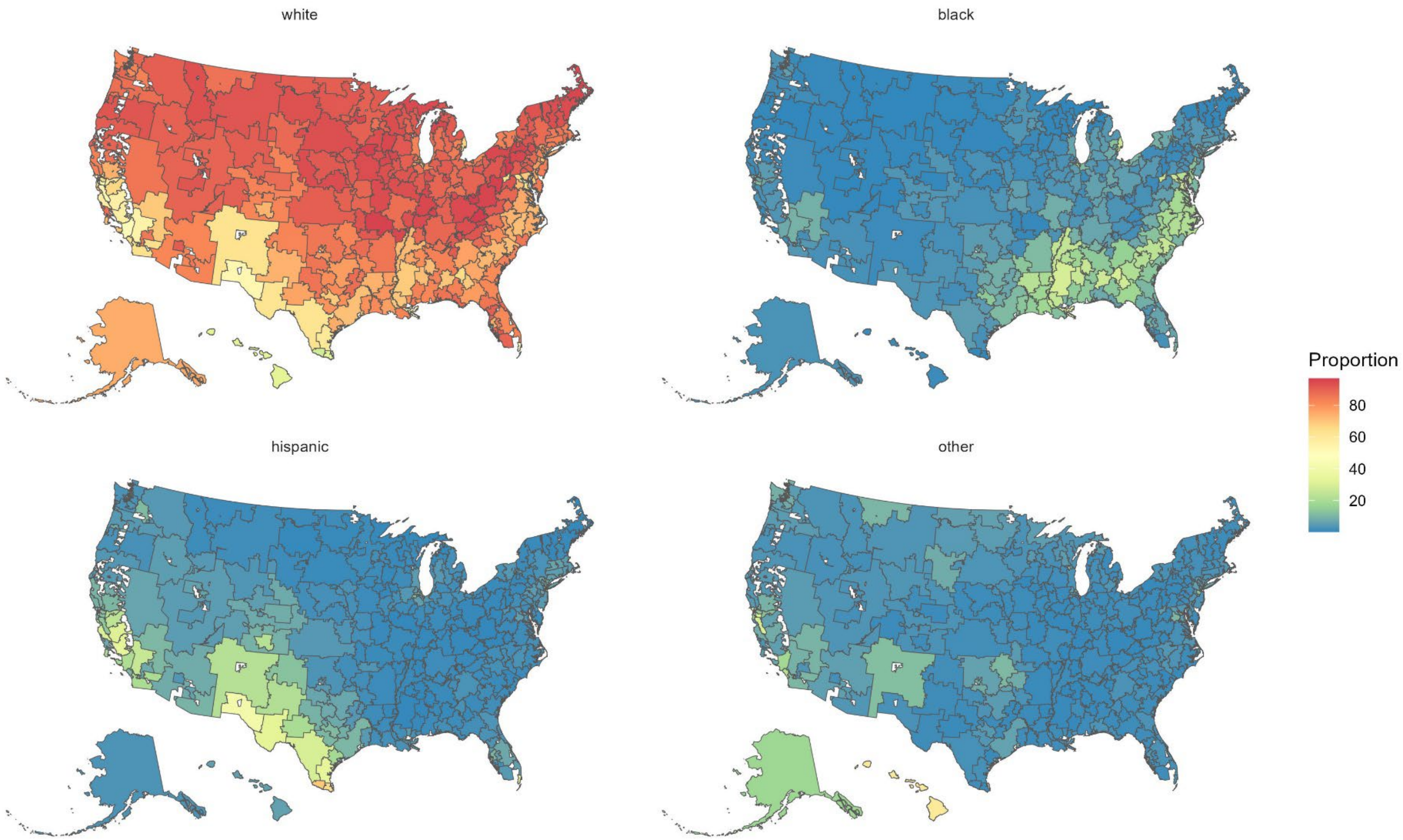

© 2024 Marcondes FO et al. *JAMA Health Forum.*

eTable 6. Number of racial and ethnic groups by hospital referral region (HRR) and mean telemedicine visits per person, second pandemic year period (March 2021 to February 2022)

	Unknown		White		Black		Hispanic		Other	
HRR	No. People	Mean	No. People	Mean	No. People	Mean	No. People	Mean	No. People	Mean
1	2105	0.6095	155244	0.57736	26211	0.60856	1577	0.48193	1339	0.44884
2	356	0.41573	32853	0.33306	5294	0.2977	484	0.36364	420	0.15
5	897	0.46154	59380	0.4472	6919	0.49834	803	0.36488	975	0.30564
6	871	0.45235	57480	0.47458	9806	0.43667	769	0.35501	876	0.31621
7	472	0.49364	23618	0.50864	9173	0.48468	242	0.44215	379	0.34565
9	206	0.25728	15993	0.48027	5723	0.40433	113	0.38053	172	0.41279
10	2185	0.81648	69833	0.80509	2369	1.06754	2574	0.73504	16248	0.87322
11	2494	0.93705	95252	0.99655	2655	1.36874	6509	0.90982	4556	0.95852
12	7233	0.95203	270151	1.01219	7156	1.34083	26043	0.90424	17569	0.89681
14	804	0.90423	42795	1.12754	829	1.58745	1595	1.09279	901	1.18979
15	2667	0.61267	96055	0.79609	2409	0.8763	11117	0.67968	3945	0.68492
16	382	0.34031	39150	0.42937	791	0.62705	751	0.36485	3504	0.39212
18	227	0.85022	28366	0.63587	821	0.84775	234	0.92735	175	0.54857
19	2217	0.61074	175393	0.54995	22280	0.69592	2034	0.55998	1814	0.53032
21	846	0.68558	51073	0.65191	381	1.03937	1833	0.40044	1523	0.53513
22	241	0.361	26988	0.4326	4476	0.53776	555	0.38559	818	0.32029
23	4657	1.79686	127144	1.70711	2877	2.02294	20827	1.51731	46939	1.77758
25	693	1.31602	47758	1.72149	2607	2.33602	28556	1.73841	4450	1.46719
31	606	1.59571	44607	1.77947	476	2.17437	4239	1.55579	1788	1.65157
33	1872	1.63835	50986	1.81618	5861	1.928	9667	1.24858	10890	1.31368
43	915	1.33005	52413	1.51253	3425	2.00029	28903	1.66664	7653	1.71789
56	16021	1.97528	262428	2.03287	39522	2.04522	109052	1.43537	96934	1.71096
58	955	1.18639	54830	1.46785	2181	1.83723	19819	1.58414	4801	1.51323
62	815	1.19632	37701	1.48633	594	1.57239	4382	1.23642	1630	1.49141
65	2294	1.99172	41259	2.35035	11782	2.61475	11275	1.80381	28185	1.88849
69	949	1.20232	39069	1.54524	912	1.90351	3529	1.55313	1381	1.37799
73	949	0.91043	65722	0.99947	557	1.17953	2944	0.97962	3108	0.83494
77	3912	1.25767	166544	1.40305	10180	1.74057	23496	1.2403	20947	1.2186
78	847	1.06139	28649	1.27324	1368	1.62573	15459	1.24381	4249	0.97341
79	1473	1.41548	63525	1.88745	10732	2.19773	29896	1.55318	10418	1.4784
80	5077	1.66594	150512	1.87297	8728	2.16567	47544	2.18482	24336	1.36091
81	3202	1.94785	57568	2.10042	9307	1.86258	11437	1.31827	31326	1.66025
82	2474	1.73605	54544	1.8591	2191	1.97398	14912	1.77582	30801	1.62946
83	894	0.9094	38389	1.19998	313	1.26518	3311	1.25249	1300	1.07
85	1877	1.62174	36640	1.74555	1203	1.88612	5512	1.28012	11487	1.24628
86	1369	1.03652	43727	1.30315	972	1.82922	12237	1.17839	2652	1.0773
87	933	1.51876	28677	1.45329	301	1.75748	7134	1.23914	1514	1.16843

© 2024 Marcondes FO et al. *JAMA Health Forum.*

89	1096	1.63777	37845	2.01379	525	2.26286	4232	1.95416	1974	1.89108
91	562	1.28826	24898	1.52052	2888	2.05506	9369	1.69719	5495	1.5869
96	1875	1.69493	57710	1.81272	1258	2.22973	15264	1.76232	6168	1.4679
101	1224	0.97059	22797	1.12427	181	1.20994	1144	0.9764	607	0.9654
102	1843	0.70863	67367	0.73861	2835	0.88395	7468	0.71706	2144	0.59795
103	6136	0.82774	154880	0.86807	6199	1.07953	14197	0.84201	5251	0.68406
104	1333	0.71043	37678	0.67819	368	0.97011	2201	0.74103	641	1.0312
105	1457	0.60535	42556	0.53424	112	1.00893	2403	0.44944	484	0.59298
106	994	0.65091	32832	0.65948	131	1.00763	3025	0.6281	401	0.9202
107	282	0.64184	12548	0.63157	228	0.76316	3821	0.60665	224	0.52679
109	2459	0.95405	43195	1.19792	3875	1.21884	3929	1.55307	2001	0.83908
110	3919	0.90712	91875	1.14425	5254	1.36563	5402	1.80618	2905	0.95559
111	4150	1.25904	103540	1.4182	5999	1.74512	6808	1.80391	3237	1.15941
112	2584	1.20395	79311	1.28794	16698	1.48102	2822	1.44614	3017	1.01989
113	8950	1.43162	197587	1.24573	83184	1.49752	9773	1.33337	15681	1.35183
115	1238	0.83199	41375	0.91275	1534	0.7764	1475	0.69966	774	0.71705
116	1308	0.7867	46934	0.72523	1110	0.67748	1637	0.85095	1025	0.60488
118	9317	1.25169	237134	1.34797	18099	1.03586	16123	1.20505	5802	1.04567
119	6074	0.66151	190163	0.69387	4424	0.61822	8711	0.7145	3274	0.59621
120	1045	0.54545	55702	0.57228	5498	0.62841	1676	0.58174	998	0.47996
122	753	0.77955	37723	0.73613	976	0.68443	2145	0.83263	698	0.60888
123	3955	0.63338	149646	0.69805	19097	0.6745	5427	0.68823	4844	0.50268
124	468	0.4359	22796	0.45556	1864	0.56009	1586	0.62989	475	0.31789
127	3116	1.37356	65962	1.53837	13821	1.22444	49812	1.69991	3460	1.25173
129	1816	0.65694	88572	0.68009	3027	0.6257	3108	0.7323	1340	0.59254
130	8089	0.78057	305660	0.8125	24375	0.85058	26889	1.01272	9563	0.71076
131	1229	0.57038	50511	0.57152	2654	0.5893	1667	0.65447	928	0.41379
133	436	0.78899	24734	0.5845	1700	0.58471	402	0.68159	595	0.27563
134	1558	0.46983	87690	0.49446	7649	0.58622	1891	0.49974	2966	0.31659
137	2854	0.85284	88897	0.83497	1408	0.71591	2001	0.67716	1341	0.65772
139	986	0.80527	33116	0.7657	2787	0.71726	1192	0.91779	976	0.56045
140	942	0.48832	49116	0.44094	11117	0.47459	1129	0.54296	758	0.31135
141	2264	0.75177	76175	0.79677	9186	0.76802	10719	0.92089	3580	0.55531
142	236	0.41949	10296	0.43959	4155	0.43249	123	0.39837	133	0.28571
144	10627	0.70603	350896	0.71785	71015	0.89285	11462	0.53804	12089	0.6175
145	970	0.3701	48148	0.34714	14519	0.37551	958	0.26514	1361	0.19985
146	304	0.71382	19397	0.71181	8887	0.69979	725	0.70897	592	0.40541
147	688	0.85029	47343	0.98122	13209	0.84359	621	0.8744	801	0.81398
148	317	0.41325	25339	0.46004	1420	0.41408	484	0.27066	212	0.60849
149	2234	0.52193	75703	0.62075	12770	0.61629	1507	0.60385	1216	0.42105
150	1583	1.28617	33414	1.34426	1132	1.12191	5751	1.10137	61053	1.20862
151	1926	0.57373	77643	0.57187	300	0.74667	4201	0.49607	1897	0.57512
152	510	0.34706	22173	0.29473	54	0.83333	928	0.25216	441	0.41043

© 2024 Marcondes FO et al. *JAMA Health Forum.*

154	428	0.74065	17813	0.71678	1243	0.92518	2649	0.7667	539	0.53618
155	1408	0.66548	57241	0.74211	17331	1.07155	4661	0.7168	1620	0.62654
156	2716	1.22275	67113	1.25792	43832	1.34977	22972	1.02877	9521	0.69867
158	1869	0.64901	68679	0.8024	1192	1.18037	4452	0.63679	3779	0.67028
161	3821	1.10547	97263	1.06527	2137	1.31212	3726	0.79549	8081	0.63482
163	1407	0.71642	37949	0.81783	1874	1.05069	1526	0.67693	4037	0.58781
164	1260	0.81111	61618	0.82239	3409	1.36052	3605	0.9165	883	0.81087
166	2796	0.68419	95524	0.79597	6989	1.09286	10847	0.80004	4812	0.63259
170	1629	0.33517	68719	0.37525	2189	0.4349	1032	0.43992	565	0.32212
171	1695	0.4472	66752	0.503	1948	0.79261	2663	0.48855	824	0.37743
172	2031	0.23831	99221	0.32294	3245	0.49337	491	0.49491	670	0.3194
173	734	0.4782	35931	0.57391	1726	0.88239	457	0.59956	527	0.50285
175	524	0.37786	17530	0.38152	543	0.59669	200	0.295	211	0.27962
179	1680	0.24524	83633	0.32106	1618	0.45612	421	0.4133	510	0.33529
180	2126	0.34948	65452	0.41106	1938	0.55986	1373	0.39548	799	0.31164
181	967	0.68046	46374	0.73977	6074	1.02601	2151	0.8331	645	0.64961
183	6928	0.5599	242240	0.57621	15499	0.87367	2989	0.64905	3501	0.53328
184	708	0.52825	21537	0.6454	283	0.98587	343	0.71429	262	0.48092
185	365	0.46301	17181	0.56394	460	0.80435	98	1.11224	119	0.58824
186	529	0.53308	22685	0.70791	3866	1.08122	3068	0.73729	541	0.63031
187	1468	0.46526	59325	0.38169	2570	0.58016	1453	0.38472	778	0.36118
188	313	0.6901	20644	0.67492	382	1.1623	120	1.18333	196	0.7449
190	824	0.37621	33260	0.35487	413	0.59322	223	0.32735	305	0.44262
191	1229	0.4109	51604	0.37685	1472	0.52174	1141	0.43646	571	0.29247
192	3120	0.37051	130666	0.34715	1580	0.53608	1566	0.28863	1606	0.34496
193	318	0.57862	13696	0.43954	190	0.95789	100	0.74	132	0.60606
194	1054	0.73245	42028	0.43128	489	0.88957	770	0.4026	513	0.48928
195	487	0.33676	24400	0.3366	68	0.44118	243	0.58436	130	0.38462
196	762	0.30709	31036	0.31457	164	0.71951	862	0.34455	770	0.32468
197	733	0.38336	23924	0.40675	592	0.57264	162	0.55556	221	0.50679
200	1145	0.47598	57421	0.48092	2033	0.59174	1989	0.5269	1182	0.40778
201	3008	0.42453	155367	0.42351	3524	0.69353	6616	0.35233	2785	0.36014
203	785	0.48662	29842	0.61263	495	0.69293	204	0.60294	290	0.41724
204	1734	0.64475	131778	0.84462	3860	0.74016	783	0.76628	951	0.54574
205	4029	0.43534	166134	0.51275	10355	0.5325	1433	0.57432	2061	0.35614
207	220	0.72727	16254	0.60016	336	0.7619	91	0.64835	100	0.41
208	537	0.5959	46472	0.53219	1581	0.67362	259	0.64093	288	0.45833
209	378	0.49471	27865	0.40635	6043	0.45507	424	0.2783	423	0.25059
210	1151	0.37706	40583	0.46039	14617	0.54074	1029	0.42663	820	0.3061
212	274	0.27007	18307	0.38395	2673	0.69847	535	0.33458	337	0.35905
213	1172	0.50853	54446	0.46676	12608	0.62793	1353	0.46933	901	0.33407
214	356	0.44382	23250	0.58275	4169	0.781	465	0.54839	261	0.64751
216	898	0.66258	24797	0.63996	3417	0.63799	1255	0.59761	553	0.3906

© 2024 Marcondes FO et al. *JAMA Health Forum.*

217	304	0.43092	22009	0.49766	5679	0.57281	185	0.60541	180	0.93889
218	879	0.74516	16111	0.76395	12679	0.67482	1243	0.59855	1035	0.35845
219	1091	0.28506	53737	0.26883	16314	0.37434	855	0.23392	724	0.22376
220	316	0.56329	14550	0.50474	1709	0.59626	439	0.68109	214	0.3271
221	1066	0.99437	49213	0.91153	198	1.43434	272	1.72426	878	1.33144
222	3651	1.09395	110046	1.01192	638	1.66144	814	1.31327	1548	0.96641
223	8512	1.33271	219925	1.29371	74072	1.41678	4677	1.32307	11806	1.01355
225	2324	1.01119	85097	0.94319	10690	1.03152	1190	1.30168	1272	0.75629
226	2975	1.5563	43021	1.75882	29549	1.70165	7260	1.22658	8804	1.4072
227	21225	2.06229	504015	1.99113	25730	2.04963	28750	2.3967	20944	1.52803
230	3065	2.28157	83607	2.10967	3551	2.31231	6644	2.57134	1564	1.97634
231	3030	1.63168	71156	1.61628	1691	1.75991	4780	2.17385	1825	1.21534
232	3215	1.42115	101707	1.3179	9438	1.82592	1861	1.17625	4074	1.04148
233	1154	2.026	31641	1.41784	2284	2.19965	1389	1.49172	1330	2.03609
234	2761	1.39334	81212	1.26001	32304	1.71121	2016	1.18998	2818	1.44145
235	769	1.21586	35727	1.18303	5373	1.79248	729	1.35802	514	1.1537
236	1685	0.79228	58281	0.74412	3161	0.97279	2540	0.69528	1427	0.55151
238	1389	0.71202	51175	0.71435	2678	1.05489	936	0.81303	844	0.78081
239	1013	1.09378	42946	1.13659	2331	1.35135	1275	1.43059	1076	0.91822
240	423	0.33333	24080	0.35801	34	0.79412	120	0.38333	765	0.55817
242	406	0.84975	17786	0.74249	1273	0.93637	465	0.77634	255	0.74118
243	543	0.67035	22149	0.58594	54	0.55556	113	0.47788	1133	0.81289
244	1066	1.01501	34837	1.15102	2826	1.70311	884	1.02149	1252	0.90335
245	3018	1.44798	49439	1.37683	8160	1.47512	655	1.37252	3688	1.25461
246	1363	0.83786	63926	0.82425	2662	1.30053	1315	1.15513	795	0.9195
248	367	0.6376	12015	0.60774	1142	0.58757	278	0.56115	266	0.94737
249	677	0.8065	27759	0.80976	76	0.61842	245	1.19592	448	1.13839
250	665	0.70226	28921	0.62432	230	1.26957	190	1.22105	1586	0.97541
251	6499	1.32236	214404	1.06323	7655	1.9373	2930	1.32799	6341	1.19161
253	1041	0.64841	44457	0.49855	426	1.26761	564	0.71099	808	0.59282
254	407	0.91155	20593	0.85238	309	1.95469	163	0.92638	298	1.06376
256	2011	1.66634	62414	1.26637	2934	2.07601	1347	1.70453	3130	1.41022
257	281	0.39146	18552	0.56727	3084	0.64235	439	0.59909	686	0.24636
258	315	0.53016	29241	0.57382	5182	0.59552	203	0.61084	211	0.46919
259	1209	0.42763	82417	0.46092	34812	0.59376	538	0.37918	873	0.37915
260	161	0.2795	17678	0.23877	6490	0.34545	83	0.45783	382	0.09424
261	194	0.29897	16629	0.23651	4856	0.29345	88	0.84091	90	0.38889
262	478	0.39331	47652	0.36941	10240	0.28887	263	0.35361	293	0.33447
263	344	0.26744	34506	0.33872	1745	0.44928	188	0.60106	174	0.21839
264	1508	0.3939	79260	0.27485	1983	0.46394	611	0.25041	644	0.22981
267	521	0.50288	41235	0.43885	257	0.84436	437	0.64531	1982	0.65035
268	5076	0.5788	215916	0.49128	12812	0.5882	4699	0.45584	3671	0.44184
270	1267	0.48382	87439	0.37643	509	0.53831	883	0.49264	901	0.36515

© 2024 Marcondes FO et al. *JAMA Health Forum.*

273	6368	0.60082	260013	0.57529	24846	0.72478	2776	0.60014	4277	0.46598
274	2491	0.43356	89480	0.39281	189	0.79365	1422	0.56188	3002	0.39907
275	471	0.22718	19198	0.28373	71	0.33803	231	0.51082	2213	0.41663
276	1732	0.62875	66385	0.50435	122	1.05738	837	0.75866	1840	0.90326
277	1863	0.36071	77435	0.37882	523	0.57361	1399	0.43531	1014	0.38166
278	4186	0.39823	142337	0.35502	3691	0.47765	3136	0.41295	1875	0.47093
279	2973	0.92936	112109	1.06259	14035	1.68999	16923	1.2473	15408	0.97443
280	1922	0.51041	86909	0.57042	1050	0.90286	6490	0.51464	4609	0.50987
281	2982	1.12978	66040	1.08763	205	1.32683	384	1.85417	687	1.25182
282	4146	1.14255	115795	1.20513	682	1.41056	1722	1.75261	1660	1.13916
283	7532	1.10462	288667	1.17085	22513	1.1978	14003	1.2216	10425	0.88758
284	3083	1.31301	82254	1.41063	5604	1.40882	14669	1.25387	10816	1.14562
285	3502	1.07196	92289	1.19338	4151	1.22982	4677	1.01689	6147	1.03921
288	2657	1.32819	68623	1.35727	6147	1.52383	6100	1.26344	12720	1.29929
289	2716	1.10862	51736	1.30944	18411	1.18771	12862	1.09998	5761	0.9757
291	754	1.17639	26308	1.20154	1898	1.07113	3234	1.15955	1312	1.00381
292	1717	1.36051	39960	1.4222	2191	1.2492	2032	1.33169	2119	1.16706
293	3593	1.00278	105431	0.97321	1864	1.01288	36823	1.00752	19887	0.86343
295	6670	1.18921	172930	1.31922	7550	1.70146	4853	1.77849	3891	1.14007
296	905	0.71602	33063	0.78317	637	0.96389	487	1.15195	609	0.81117
297	1470	1.27619	17328	1.54559	16474	1.574	15449	1.77824	2513	1.3072
299	1998	0.83784	65417	0.94301	7104	1.11655	2000	1.2705	2194	0.81449
300	912	0.83004	28416	0.91522	803	1.14819	517	0.99613	457	0.81619
301	16684	1.33427	345774	1.46276	37577	1.37259	29611	1.4372	32352	1.25544
303	15253	1.97836	217585	2.19083	50587	1.62536	33891	1.83435	25566	1.71458
304	2384	1.23112	59178	1.2495	5151	1.22539	2549	1.38839	1638	1.04579
307	2956	0.79635	84366	0.99175	2560	1.22617	1009	1.26065	1605	0.85047
308	4280	1.35374	88676	1.47816	10151	1.66476	9088	1.52509	4751	1.03115
309	2441	0.85293	90770	0.77671	1814	1.06229	1092	0.84341	1912	0.41632
311	4523	0.66659	177723	0.62086	28059	0.78845	4441	0.66764	4572	0.48863
312	2701	0.91485	99437	0.76026	26270	0.6917	2119	0.71449	4725	0.65608
313	743	0.80619	29720	0.81541	6569	0.97975	724	0.5511	774	0.46124
314	1551	0.48291	81722	0.49365	23043	0.4407	1590	0.71509	1337	0.51234
315	396	0.62121	25603	0.4003	1030	0.45534	397	0.35013	550	0.29273
318	3588	0.80128	132461	0.72643	39290	0.73441	4734	0.74778	5187	0.59553
319	1836	0.66231	64034	0.66583	7335	0.64717	796	0.86432	787	0.65693
320	2002	0.57493	79064	0.55248	7790	0.8172	1537	0.5257	1162	0.50258
321	488	0.16598	29386	0.19189	88	0.48864	151	0.25828	1320	0.32121
322	1258	0.87043	58249	0.4442	339	0.97345	447	0.65324	2979	0.85364
323	377	0.91247	19102	0.48053	113	1.12389	279	0.56631	1179	0.50636
324	330	0.49697	16261	0.24445	134	0.37313	176	0.36364	975	0.37949
325	1642	0.69671	41415	0.7618	3238	1.00649	320	0.71875	639	0.59468
326	1799	0.51251	43468	0.57401	1312	0.88034	320	0.77188	425	0.59294

© 2024 Marcondes FO et al. *JAMA Health Forum.*

327	5615	0.50009	112946	0.58589	9168	0.61726	739	0.53451	1842	0.47611
328	6288	0.89281	149330	0.81542	18628	0.93596	2956	0.9753	3260	0.82301
329	7893	0.69074	229783	0.71085	13377	0.86566	1676	0.93974	3731	0.60574
330	3310	0.44773	86380	0.48236	6944	0.63537	693	0.46176	1123	0.39092
331	787	0.92757	21057	0.80762	899	1.00222	970	0.84124	288	0.75
332	1548	0.56783	33854	0.5711	1257	0.68894	303	0.76238	677	0.43427
334	3919	0.43098	82364	0.49151	4272	0.57748	2070	0.57053	1024	0.40137
335	1263	0.57641	49242	0.58182	2834	0.77488	625	0.6496	419	0.68496
336	193	0.35233	20725	0.34466	2019	0.48093	1140	0.44035	2143	0.38171
339	2593	0.61627	185345	0.59783	9928	0.68503	5902	0.54727	12705	0.5771
340	1702	0.46298	120213	0.57076	6074	0.76819	2344	0.51706	15543	0.59339
341	1034	0.80561	36890	0.84741	89	0.89888	893	0.84546	951	1.08307
342	1704	0.95657	75769	0.80501	392	0.92857	2024	0.86166	2041	0.84615
343	1278	0.82316	66120	0.90041	327	0.87768	2653	0.82247	2225	0.85753
344	5374	1.13268	174084	1.0302	2948	1.23813	8066	0.80523	8949	0.89664
345	403	0.75682	18176	0.98625	158	1.41772	1706	0.77726	760	1.025
346	3726	0.81857	132510	0.87407	4136	1.18206	6794	1.25243	2660	0.80489
347	409	0.72127	26068	0.61044	166	1.33735	115	1.04348	131	0.63359
350	1163	0.55202	51950	0.64293	546	1.10256	445	1.0427	420	0.65714
351	1281	0.70023	66367	0.64289	1025	0.82146	670	1.00896	488	0.57377
352	3060	0.68399	96639	0.63996	3235	0.77589	1763	0.93988	1560	0.5859
354	258	0.78295	14035	0.67004	251	0.95219	105	0.89524	97	0.90722
355	1918	0.73514	63909	0.63597	1851	0.91842	1869	0.81969	1050	0.60952
356	11863	1.15241	299017	1.15953	49047	1.32277	10365	1.59875	14495	1.05264
357	3946	0.85606	179880	0.77944	7873	0.91655	1181	0.89246	1984	0.79486
358	1772	0.64221	59815	0.71892	1380	1.17319	2482	1.25342	694	0.62968
359	554	0.73466	21297	0.59675	115	0.86087	120	0.83333	178	0.57303
360	887	0.91995	42958	1.06162	576	1.76215	891	1.27834	483	0.96066
362	497	1.16298	29410	1.02421	520	1.18846	598	1.29264	297	0.79798
363	1309	0.63178	42149	0.64644	1239	0.87732	841	0.95125	467	0.59529
364	3359	1.41739	89568	1.45302	2524	1.25792	4466	1.45231	2378	1.21867
365	3797	0.53779	140593	0.58676	20147	0.65444	2037	0.6485	2186	0.45517
366	2401	0.36526	103854	0.40722	33125	0.47915	1371	0.44785	1546	0.39133
367	401	0.41646	32042	0.37267	11813	0.41505	290	0.34483	271	0.47232
368	2380	0.55336	92508	0.65062	7797	0.74003	1204	0.60963	1142	0.55342
369	698	0.2851	36020	0.30752	4011	0.30117	433	0.37413	477	0.28302
370	712	0.55758	30605	0.33942	127	0.20472	372	0.25806	2752	0.5436
371	2219	0.40153	102516	0.32885	456	0.45833	898	0.50557	3203	0.47924
373	1076	0.62361	65333	0.73857	3923	0.92633	588	0.80102	763	0.55963
374	398	0.30653	37751	0.2477	3941	0.36894	240	0.23333	231	0.35065
375	479	0.44259	25003	0.43575	287	0.45993	210	0.48571	178	0.20787
376	559	0.37567	45972	0.51299	420	0.43333	162	0.40741	251	0.45418
377	2795	0.40644	132094	0.45307	3248	0.51324	1087	0.48482	1285	0.4428

© 2024 Marcondes FO et al. *JAMA Health Forum.*

379	1978	0.41658	127810	0.42287	43563	0.48601	1444	0.42036	2180	0.28945
380	5182	0.47202	242926	0.48443	18014	0.52598	3428	0.47229	3452	0.41078
382	333	0.43243	30853	0.48504	829	0.6152	3352	0.45644	424	0.37264
383	484	0.3657	36562	0.3819	844	0.42062	5540	0.413	729	0.27846
385	3434	1.03378	118085	1.11815	6776	1.22683	15429	0.91043	5062	0.89708
386	293	0.75768	28669	0.87753	4872	1.15374	1559	0.58884	543	0.69797
388	329	0.4924	18552	0.50986	1888	0.68697	1973	0.39432	347	0.51873
390	335	0.67463	21174	0.72155	766	0.8342	12122	1.05956	759	0.72991
391	7206	0.97002	285791	1.05597	31571	1.67676	24980	0.95556	16871	1.25245
393	763	0.78244	34001	1.02379	2131	0.95683	26122	0.95085	1550	0.81677
394	2391	0.78628	121439	0.8713	11000	1.32255	11403	0.79926	4334	0.89663
396	150	0.75333	8815	0.87975	114	1.01754	17346	1.85288	317	1.18297
397	6850	0.74511	285717	0.9073	43919	1.1807	41350	0.76793	23813	0.74673
399	240	0.37083	18719	0.31252	2130	0.40282	598	0.19398	196	0.5102
400	641	0.30109	44238	0.38872	1671	0.42968	12803	0.39022	762	0.31759
402	134	0.94776	6681	1.15791	96	1.22917	16477	2.5308	344	1.22093
406	306	0.66013	16445	0.59538	776	0.7384	8519	0.57894	356	0.44663
411	173	0.3237	13928	0.40717	300	0.59333	3637	0.41518	176	0.41477
412	3185	0.91429	140667	0.94958	10935	1.01427	66056	1.22092	4840	0.78905
413	588	0.4881	29808	0.6101	6105	0.60721	4869	0.52475	1806	0.40587
416	685	0.40146	55315	0.36111	4280	0.45397	2354	0.25828	552	0.34239
417	217	0.45622	12037	0.7148	628	0.88057	3061	0.78634	151	0.89404
418	378	0.53704	24933	0.53664	2692	0.61441	2339	0.44036	352	0.55398
420	272	0.63971	22124	0.70851	1104	0.99819	1733	0.86901	476	0.7542
421	482	0.57884	29002	0.66426	332	0.75301	1870	0.65027	863	0.42874
422	622	0.87138	31296	0.66638	76	1.10526	1185	0.58819	644	0.63975
423	3755	0.57204	151783	0.53753	706	0.78187	7988	0.4523	5234	0.49178
424	3254	1.23694	85566	1.21184	506	1.28854	739	1.33829	1729	1.12261
426	6731	1.04591	133213	1.15476	15031	1.16646	9891	0.87413	19826	1.33829
427	2858	0.48915	79458	0.53852	5030	0.44573	928	0.58513	1170	0.49145
428	866	0.42956	33062	0.36111	4698	0.36356	234	0.65385	315	0.26984
429	1416	0.56144	53741	0.54124	13909	0.56618	1003	0.67797	1746	0.41638
430	2701	0.6953	102885	0.76233	28484	0.78823	3057	0.69382	5024	0.41123
431	4769	0.723	154525	0.72735	38695	0.75508	2720	0.85515	4212	0.55627
432	1840	0.4413	85632	0.46402	4360	0.48876	437	0.74371	782	0.36061
435	1171	0.7199	56754	0.76305	1967	0.90951	814	0.85012	691	0.76122
437	1795	0.70362	59276	0.72836	577	0.84055	1840	0.56033	3111	0.56959
438	1120	0.66071	46611	0.65285	802	0.58479	1304	0.63344	2624	0.49466
439	7970	0.89724	220260	0.88246	7198	1.01723	6814	0.7354	23459	0.64517
440	4500	0.56911	199991	0.51996	1248	0.80849	8674	0.5392	6103	0.56546
441	1636	0.83313	61383	0.81472	4028	0.84558	2377	0.71939	5898	0.63835
442	456	0.5	28078	0.46716	205	0.98049	3706	0.44765	1273	0.76198
443	931	0.80988	89994	0.79272	2117	0.90458	367	0.80109	657	0.64688

© 2024 Marcondes FO et al. *JAMA Health Forum.*

444	393	0.75318	36523	0.83712	535	0.79439	104	0.57692	220	0.80455
445	541	0.79667	39129	0.61394	454	0.78194	214	0.56542	270	0.41111
446	686	0.47522	18566	0.42082	103	0.63107	276	0.63406	1032	0.38275
447	1371	0.32385	40961	0.37416	258	1.03876	554	0.54513	1399	0.55111
448	886	0.58014	39229	0.55296	204	0.72549	258	0.91473	613	0.54649
449	4455	0.89989	113816	0.77814	2149	1.09027	1639	0.93594	1956	0.82106
450	1140	0.32895	39822	0.43112	100	0.73	323	0.74303	935	0.53904
451	7471	0.58653	171256	0.672	13397	0.94073	9233	0.72728	5255	0.59543
452	570	0.45789	15170	0.46454	108	0.74074	217	0.70507	311	0.31511
456	652	0.40798	19289	0.45373	73	0.43836	106	0.53774	725	0.40414
457	755	0.22384	32769	0.33861	129	0.65891	1179	0.43172	1163	1.33534

© 2024 Marcondes FO et al. *JAMA Health Forum.*

eTable 7. Odds of having a telemedicine visit (video or audio), pandemic second year period – logistic regression model results

	Telemedicine (audio-video) visits (%)		
	OR (95% CI)		
**Race/ethnicity**			
White	Ref	Ref	Ref
Black	1.104 (1.099, 1.108)	0.965 (0.960, 0.969)	0.916 (0.912, 0.921)
Hispanic	1.274 (1.268, 1.280)	1.257 (1.251, 1.264)	0.856 (0.852, 0.861)
Other	1.278 (1.271, 1.285)	1.388 (1.380, 1.396)	0.931 (0.926, 0.937)
**Regression model**	Model 1<br><br>No other variables	Model 2<br><br>Age, Documented sex, Clinical indicators	Model 3<br><br>Adding indicator variables for HRR[a]

a Abbrev: HRR - Hospital Referral Region

© 2024 Marcondes FO et al. *JAMA Health Forum.*

eTable 8. Telemedicine visits counts per 100 individuals by race and ethnicity, pandemic second year period – Sensitivity analysis restricting to patients with at least 1 visit in the year

	Telemedicine (audio-video) visits (%)		
	Estimate (95% CI)		
Race/ethnicity			
White	Ref	Ref	Ref
Black	28.0 (27.4, 28.7)	2.3 (1.6, 2.9)	-3.5 (-4.2, -2.8)
Hispanic	54.7 (54.0, 55.5)	34.4 (33.7, 35.2)	-6.8 (-7.5, -6.0)
Other	30.2 (29.3, 31.0)	36.4 (35.6, 37.3)	-7.5 (-8.3, -6.6)
Regression model	Model 1<br><br>No other variables	Model 2<br><br>Age, Documented sex, Clinical indicators	Model 3<br><br>Adding indicator variables for HRR[a]

a Abbrev: HRR - Hospital Referral Region

© 2024 Marcondes FO et al. *JAMA Health Forum.*

eTable 9. Telemedicine visits counts per 100 individuals by race and ethnicity, pandemic second year period – Sensitivity analysis restricting to mental health telemedicine visits

	Telemedicine (audio-video) visits (%)		
	Estimate (95% CI), per 100 individuals		
Race/ethnicity			
White	Ref	Ref	Ref
Black	1.0 (0.5, 1.4)	-9.3 (-9.7, -8.8)	-11.7 (-12.2, -11.3)
Hispanic	0.8 (0.3, 1.4)	-7.0 (-7.5, -6.5)	-12.1 (-12.7, -11.6)
Other	-10.8 (-11.4, -10.2)	-4.6 (-5.2, -4.0)	-11.4 (-12.0, -10.8)
Regression model	Model 1<br><br>No other variables	Model 2<br><br>Age, Documented sex, Clinical indicators	Model 3<br><br>Adding indicator variables for HRR[a]

a Abbrev: HRR - Hospital Referral Region

© 2024 Marcondes FO et al. *JAMA Health Forum.*

eFigure 6. Total visit counts by race and ethnicity group, relative to White beneficiaries, 2019 and pandemic second year period (March 2021 to February 2022)[a]

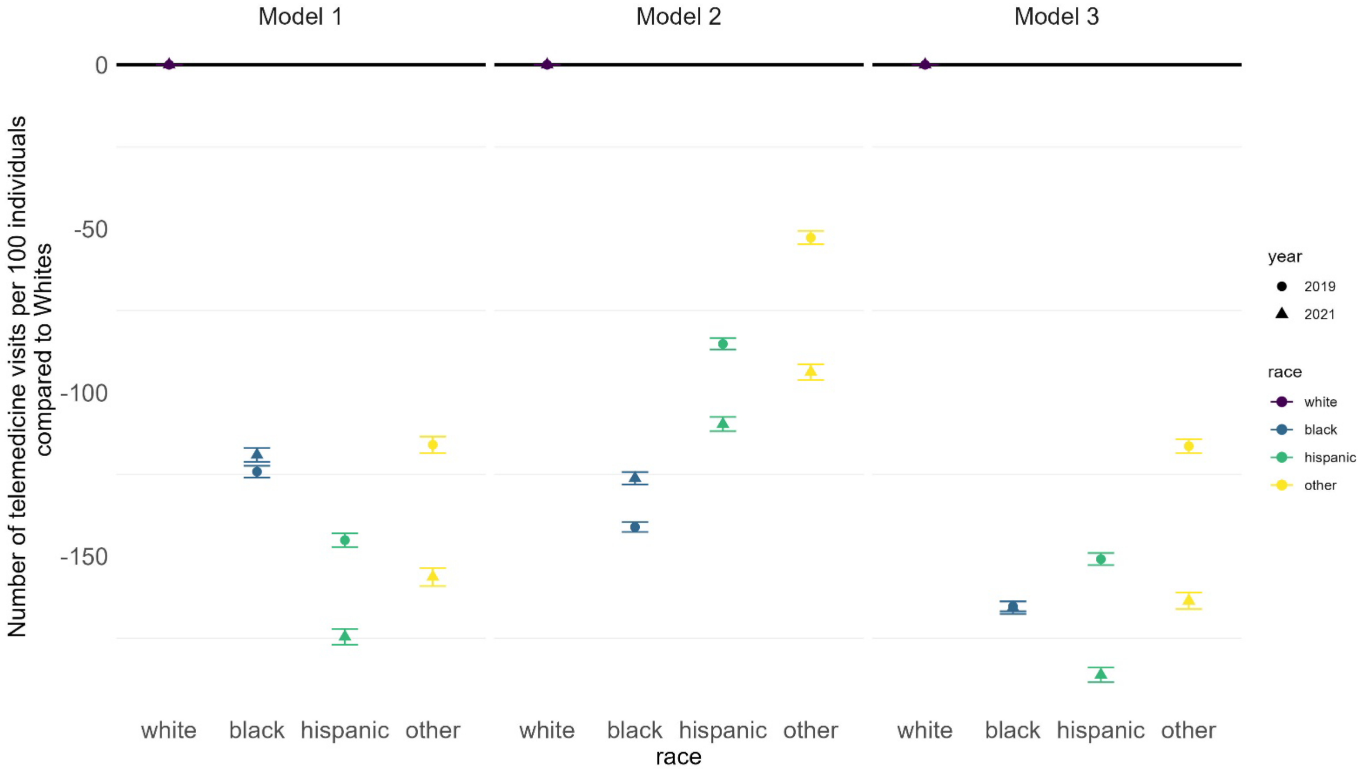

a Note: bars above and below the dots refer to the 95% CIs

© 2024 Marcondes FO et al. *JAMA Health Forum.*
