# Supplementary material for: Racial and Ethnic Differences in Telemedicine Use
Source: JAMA Health Forum. 2024 Mar 22;5(3):e240131. doi: 10.1001/jamahealthforum.2024.0131 (PMC10960201; doi:10.1001/jamahealthforum.2024.0131)
Supplement: Supplement 2. — Data sharing statement [file jamahealthforum-e240131-s002.pdf]

Data Sharing Statement

Marcondes. Racial and Ethnic Differences in Telemedicine Use. *JAMA Health Forum*.
Published March 22, 2024. doi:10.1001/jamahealthforum.2024.0131

Data
Data available: No

Additional Information
Explanation for why data not available: Data is proprietary to the Centers for Medicare and Medicaid Services and cannot be shared publicly.
